# Conservation, loss, and redeployment of Wnt ligands in protostomes: implications for understanding the evolution of segment formation

**DOI:** 10.1186/1471-2148-10-374

**Published:** 2010-12-01

**Authors:** Ralf Janssen, Martine Le Gouar, Matthias Pechmann, Francis Poulin, Renata Bolognesi, Evelyn E Schwager, Corinna Hopfen, John K Colbourne, Graham E Budd, Susan J Brown, Nikola-Michael Prpic, Carolin Kosiol, Michel Vervoort, Wim GM Damen, Guillaume Balavoine, Alistair P McGregor

**Affiliations:** 1 Department of Earth Sciences, Palaeobiology, Villavägen 16, SE-75236 Uppsala, Sweden; 2 Centre de Génétique Moléculaire du CNRS, FRE 3144, avenue de la Terrasse 91198 Gif-sur-Yvette, France; 3 Georg-August-Universität Göttingen, Johann-Friedrich-Blumenbach-Institut für Zoologie und Anthropologie, Abteilung Entwicklungsbiologie, GZMB, Ernst-Caspari-Haus, Justus-von-Liebig-Weg 11, 37077 Göttingen, Germany; 4 Department of Integrative Biology, University of California, Berkeley, CA 94720, USA; 5 Division of Biology, Kansas State University, Manhattan, KS 66506, USA; 6 Department of Organismic and Evolutionary Biology, Harvard University, 16 Divinity Ave, Cambridge MA 02138, USA; 7 Institut für Populationsgenetik, Veterinärmedizinische Universität Wien, Veterinärplatz 1, A-1210, Vienna, Austria; 8 The Center for Genomics and Bioinformatics, Indiana University, Bloomington, IN 47405, USA; 9 Friedrich-Schiller-University Jena, Department of Genetics, Philosophenweg 12, 07743 Jena, Germany; 10 Institut Jacques Monod, CNRS/Université Paris-Diderot, 15 rue Hélène Brion, 75205 Paris Cedex 13, France; 11 Genzyme Corporation, One The Mountain Road, Framingham, MA 01701, USA; 12 Monsanto Company, St. Louis, MO, 63107, USA

## Abstract

**Background:**

The *Wnt *genes encode secreted glycoprotein ligands that regulate a wide range of developmental processes, including axis elongation and segmentation. There are thirteen subfamilies of *Wnt *genes in metazoans and this gene diversity appeared early in animal evolution. The loss of *Wnt *subfamilies appears to be common in insects, but little is known about the *Wnt *repertoire in other arthropods, and moreover the expression and function of these genes have only been investigated in a few protostomes outside the relatively *Wnt*-poor model species *Drosophila melanogaster *and *Caenorhabditis elegans*. To investigate the evolution of this important gene family more broadly in protostomes, we surveyed the *Wnt *gene diversity in the crustacean *Daphnia pulex*, the chelicerates *Ixodes scapularis *and *Achaearanea tepidariorum*, the myriapod *Glomeris marginata *and the annelid *Platynereis dumerilii*. We also characterised *Wnt *gene expression in the latter three species, and further investigated expression of these genes in the beetle *Tribolium castaneum*.

**Results:**

We found that *Daphnia *and *Platynereis *both contain twelve *Wnt *subfamilies demonstrating that the common ancestors of arthropods, ecdysozoans and protostomes possessed all members of all *Wnt *subfamilies except *Wnt3*. Furthermore, although there is striking loss of *Wnt *genes in insects, other arthropods have maintained greater *Wnt *gene diversity. The expression of many *Wnt *genes overlap in segmentally reiterated patterns and in the segment addition zone, and while these patterns can be relatively conserved among arthropods and the annelid, there have also been changes in the expression of some *Wnt *genes in the course of protostome evolution. Nevertheless, our results strongly support the parasegment as the primary segmental unit in arthropods, and suggest further similarities between segmental and parasegmental regulation by *Wnt *genes in annelids and arthropods respectively.

**Conclusions:**

Despite frequent losses of *Wnt *gene subfamilies in lineages such as insects, nematodes and leeches, most protostomes have probably maintained much of their ancestral repertoire of twelve *Wnt *genes. The maintenance of a large set of these ligands could be in part due to their combinatorial activity in various tissues rather than functional redundancy. The activity of such *Wnt *'landscapes' as opposed to the function of individual ligands could explain the patterns of conservation and redeployment of these genes in important developmental processes across metazoans. This requires further analysis of the expression and function of these genes in a wider range of taxa.

## Background

Wnt signalling regulates many developmental processes in metazoans, including cell proliferation, migration and pattern formation [1]. The *Wnt *genes encode secreted glycoprotein ligands that bind to various transmembrane receptors thereby triggering intracellular cascades, including the β-catenin pathway, to regulate transcription in target cells [2].

Among protostomes, Wnt signalling has been most intensively studied in the nematode worm *Caenorhabditis elegans *and the fly *Drosophila melanogaster*. These two model ecdysozoans have five and seven *Wnt *genes respectively [3-13], which generally reflects the number of *Wnt *genes found in insects with sequenced genomes [14-16]. However, thirteen subfamilies of *Wnt *genes have been reported in metazoans [17-19]. All thirteen subfamilies are found in deuterostomes, although *WntA *may have been lost in vertebrates and other lineages [18-20]. Twelve subfamilies have also been recently reported in lophotrochozoans, which is evidence for a large set of *Wnt *genes ancestrally in protostomes [17]. This complexity in the repertoire of *Wnt *genes appeared very early in metazoan evolution because twelve subfamilies are also found in the cnidarians *Nematostella vectensis *and *Hydra magnipapillata *[18,21,22]. Taken together, these earlier studies demonstrate striking patterns of *Wnt *gene loss in insects and *Caenorhabditis *in comparison to other animals. However, it is not yet known if this loss of *Wnt *genes is a derived feature of insects or a more general characteristic of arthropods (or ecdysozoans). Moreover, our understanding of the evolution of the *Wnt *gene family is hampered by the paucity of expression and functional studies in arthropods and protostomes other than *Drosophila *and *Caenorhabditis *[14,17,23-25].

A major exception to this paucity of knowledge is *wingless *(*wg/Wnt1*). Among many other roles in *Drosophila*, *wg *functions as a segment polarity gene to specify and maintain boundaries and cell fates across the primary segmental units or parasegments [3,9,26-29]. *wg *is expressed at the posterior boundary of each parasegment directly juxtaposed to cells expressing *engrailed (en) *at the anterior parasegmental boundary. Studies of *wg *and *en *in other arthropods indicate that their delineation of parasegmental boundaries is an ancestral feature of these animals [30-34]. Furthermore, the expression of the *wg*, *en *and *hh *homologues also delimits segmental boundaries in the annelid, *Platynereis dumerilii*: a representative of lophotrochozoans, the large sister-clade of the ecdysozoans within protostomes [35,36]. This observation suggests that the *wg-en *regulatory system was either independently recruited for segment boundary determination in annelids and arthropods or is plesiomorphic with respect to a segmented common ancestor. This debate [37-39] could be resolved by comparing the expression of other genes involved in segmentation within and among arthropods and annelids. Intriguingly, segmental expression of several other *Wnt *genes has been observed in various arthropods, suggesting that these *Wnt *genes may also be involved in segmentation [23,24,30,33].

To investigate the *Wnt *repertoire of arthropods and protostomes more broadly, we surveyed the *Wnt *genes found in a crustacean, the water flea *Daphnia pulex*, a myriapod, the millipede *Glomeris marginata*, two chelicerates, the spider, *Achaearanea tepidariorum *and the tick, *Ixodes scapularis*, and an annelid, the polychaete worm *Platynereis dumerilii*. We then characterised the expression of *Wnt *genes in *Achaearanea*, *Glomeris*, *Platynereis*, and *Tribolium *to compare the possible roles of *Wnt *genes in segmentation and other developmental processes among the arthropods, and protostomes generally.

Our survey and analysis of *Wnt *genes demonstrates that the common ancestor of arthropods contained twelve of the thirteen subfamilies, and, therefore, that the ancestral protostome contained all *Wnt *gene subfamilies except *Wnt3 *as was previously suggested by data from lophotrochozoans [17]. We found twelve, eleven and ten *Wnt *genes in *Daphnia, Achaearanea and Ixodes *respectively, including orthologues of *Wnt2 *and *Wnt4*, which are not found in insects. This shows that the loss of *Wnt *genes observed in insects is not a general feature of arthropods.

We found that many *Wnt *genes are expressed in segmentally reiterated patterns in protostomes. For example, *Wnt10 and Wnt16*, and *wg*, are expressed in similar segmental patterns in arthropods and in the annelid *Platynereis*. However, we also found taxon-specific segmental expression of several *Wnt *genes, even among the arthropods. This pattern of conservation and redeployment of *Wnt *genes expressed in segmentally reiterated patterns in arthropods and the annelid was also reflected in the expression of these genes in the posterior segment addition zone (SAZ) [31,40,41] (hereafter we use this more general term rather than 'growth zone' as commonly used for arthropods, see Discussion), appendages, nervous system and other tissues. Indeed, the overlapping expression of multiple *Wnt *genes in the same tissues supports the hypothesis that Wnt signalling operates through a combinatorial code of different Wnt ligands [42].

## Methods

### Wnt gene sequences

Members of the *Wnt *subfamilies, 1, 2, 5, 7, 8, A and 16, were previously isolated from *Achaearanea *or *Cupiennius*, and subfamilies 1, A and 16 from *Glomeris *(Additional file 1). Note that *Glomeris Wnt16 *and *WntA *were previously erroneously characterised as *Wnt7 *and *Wnt5 *orthologues respectively [33]. We obtained sequences of a further four *Wnt *genes from both *Achaearanea *and *Glomeris *using degenerate PCR with embryonic cDNA template. The sequences of degenerate primer pairs used to isolate *Wnt *genes are shown in Additional file 2. Larger fragments of initial PCR fragments were obtained via RACE PCR using the Marathon RACE Kit (Clontech). RNA isolation from spiders and *Glomeris*, and cDNA synthesis was carried out as described previously [33,43].

For *Daphnia*, known *Wnt *gene sequences were obtained from GenBank and protein sequences were used to perform tblastn searches of assembled genomic scaffolds, predicted gene models and ESTs (*Daphnia pulex *v1.1, September 2006; http://www.jgi.doe.gov/Daphnia and http://wFleaBase.org). Segment pairs with an E-value smaller than 10^5 ^were selected and the corresponding scaffolds were manually curated with the help of Dappu v1.1 filtered gene models. Predicted gene structures were refined by comparison to *Wnt *genes from other species (*Nematostella vectensis*, *Drosophila melanogaster*, *Tribolium castaneum*, *Apis mellifera*, *Homo sapiens*, *Mus musculus *and *Strongylocentrotus pupuratus*) to identify the correct open reading frames. Partial cDNAs were cloned to confirm most intron-exon boundaries. Briefly, TRIzol (Invitrogen) was used to isolate RNA from *Daphnia *embryos of mixed stages. RNA was reverse transcribed using SuperScriptIII (Invitrogen) and RT-PCR was performed using primers specific to each predicted *Wnt *open reading frame. Sequence from each gene model was used to search the *Daphnia *assembly and confirm the presence of twelve *Wnt *gene sequences and the absence of any additional *Wnt *family members (Additional file 1). The synteny of *Daphnia *Wnt genes was inferred from their linkage on the same genomic scaffolds.

Gene models of nine *Wnt *genes from the tick *Ixodes scapularis *were retrieved from VectorBase [44] and GenBank deposits (Additional file 1). A fragment of a tenth *Ixodes Wnt *gene was also identified through tblastn searches (Additional file 1).

Six *Platynereis Wnt *genes were isolated in a previous study [19]. Two more *Wnt *genes (*Pd-Wnt5 *and *Pd-Wnt8*) were found in an EST collection [45]. To identify remaining *Wnt *orthologues a combination of more specific primers were used (Additional file 2). The accession numbers of all *Wnt *gene sequences used in this study are shown in Additional file 1.

### Phylogenetic analysis

Two data sets were used for the analysis, the first set consisted of 93 amino acid sequences from arthropods, *Platynereis *and human (Additional file 3: Wnt sequence data set 1), and the larger second set included additional sequences from a nematode, cnidarian and three lophotrochozoans (Additional file 4: Wnt sequence data set 2). Sequences in both data sets were aligned using T-coffee [46] and hand-edited in SeaView [47] to remove poorly aligned amino acid positions (Additional files 3 and 4).

Initially, the best-scoring substitution model was determined among the amino acid models in RAxML [48] as WAG+F+Γ (WAG with empirical base frequencies and the Γ model of rate heterogeneity; Whelan and Goldman [49]).

Bayesian phylogenetic analyses were performed with MrBayes [50]. The final topology was estimated using 13,000,000 iterations using 3250 burning cycles and sampling every 1000 iteration. Clade support was assessed with posterior probabilities computed with MrBayes and non-parametric bootstrapping implemented in RAxML [48] based on 1000 replicates.

### Animals

Spiders (*Achaearanea tepidariorum *and *Cupiennius salei*) were obtained from laboratory stocks in Cologne and Göttingen [24,51]. Spider embryos were staged according to Akiyama-Oda and Oda [52]. General handling and staging of *Glomeris *is described in Janssen et al., [33]. *Tribolium *beetles (Ga-1 strain) were obtained from laboratory stocks at Kansas State University. Beetles were reared at 30°C in whole-wheat flour supplemented with 5% dried yeast. *Platynereis *larvae and juveniles were obtained from a breeding culture established in Gif-sur-Yvette according to the protocols of Fischer and Dorresteijn http://www.platynereis.de.

### Staining and microscopy

Whole mount in situ hybridisation (WMISH) was performed for spiders as described in the published protocol for *Cupiennius *embryos [43]. For *Glomeris*, WMISH was performed as described in Prpic and Tautz [53] and Janssen et al. [32]. Both spider and *Glomeris *embryos were counterstained with Sytox Green or DAPI and images were captured with a Leica dissection microscope or a Zeiss Axioplan-2 microscope. For *Tribolium *and *Platynereis*, WMISH was performed as described previously [41,54-57]. All digital images have been subjected to adjustment of brightness, colour values and contrast using Adobe Photoshop CS3.

In *Achaearanea *and *Cupiennius *gene expression was investigated in stage 4 to stage 10 embryos, which represent germ disc embryos with radial symmetry (stages 4 to 6), and germ band embryos with axial symmetry (stages 7 to 10) and up to 7 opisthosomal segments [52,58]. In *Glomeris*, gene expression was investigated in stage 0 (blastoderm) to stage 6.1 embryos; see Janssen et al. [33] for a detailed description of staging. In *Tribolium*, gene expression was analysed in embryos at the fully extended germ band stage. In *Platynereis*, as in many other annelids, the elongation of the body axis continues during post-embryonic development as new segments are added from a sub-terminal SAZ [41]. We thus compared the expression of *Wnt *genes during trunk formation in both embryonic and post-embryonic development.

## Results

### Phylogenetic analysis of Wnt protein sequences and designation of Wnt gene subfamilies

**Figure 1:**
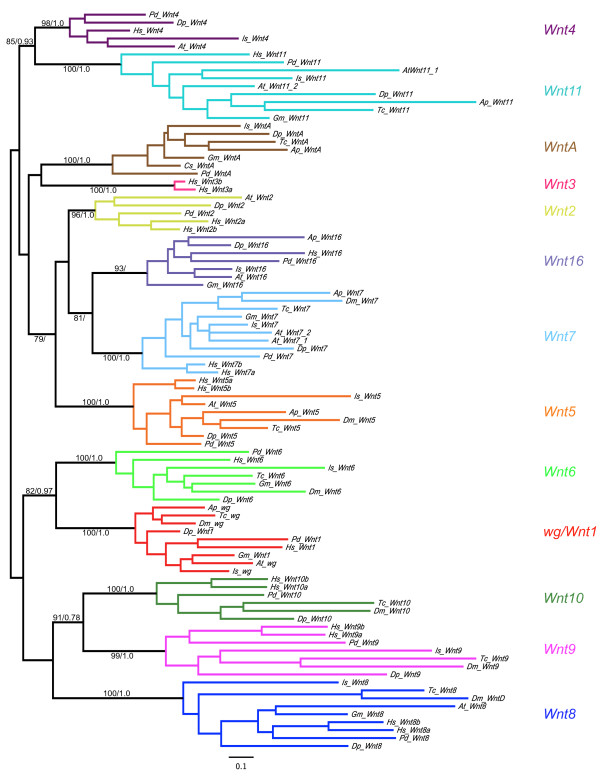
**Maximum likelihood tree of Wnt amino acid sequences in selected metazoans**. Bootstrap values/poster probabilities from Maximum likelihood and Bayesian analyses respectively are given on branches. Note that support for the position of *Is_Wnt16 *was only found using Maximum likelihood (see also Additional file 5). Wnt amino acid sequences were used from the following species: *Achaearanea tepidariorum (At), Acyrthosiphon pisum (Ap), Cupiennius salei (Cs), Daphnia pulex (Dp), Drosophila melanogaster (Dm), Glomeris marginata (Gm), Homo sapiens (Hs), Ixodes scapularis (Is), Platynereis dumerilii (Pd) *and *Tribolium castaneum (Tc)*. Bootstrap values and posterior probabilities of all branches are given in Additional files 6 and 7 respectively.

Combining the findings of database searches, genome annotation, degenerate PCR and *Wnt *genes identified in previous studies (see Methods), we found a total of eleven *Wnt *genes in *Achaearanea*, (with *WntA *from *Cupiennius *representing a twelfth spider *Wnt *gene), seven in *Glomeris*, twelve in *Daphnia*, ten in *Ixodes *and twelve in *Platynereis*. These sequences were then aligned with the Wnt sequences of *Acyrthosiphon pisum*, *Drosophila*, *Homo *and *Tribolium *(Additional files 1 and 3). A further alignment was generated using a larger set of *Wnt *genes containing the Wnt sequences from *Caenorhabditis, Capitella, Helobdella, Lottia *and *Nematostella *in addition to the sequences used in the first set of *Wnt *genes (Additional files 1 and 4). We then carried out phylogenetic analyses using Maximum likelihood approaches (Wnt sequence sets 1 and 2) and additional Bayesian approaches (Wnt sequence set 1) (see Methods) (Figure 1 and Additional files 5, 6, 7).

**Figure 2:**
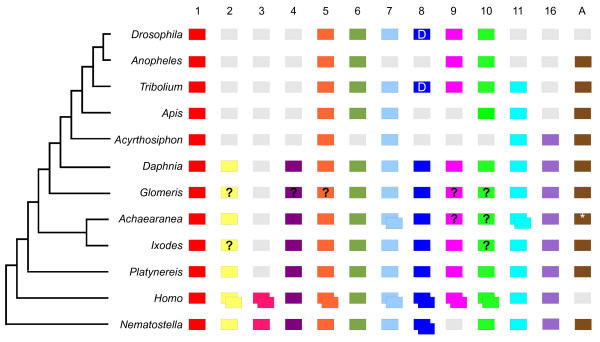
**Metazoan *Wnt *genes**. The *Wnt *subfamilies (1 to 11, 16 and A) found in the various metazoans are represented by coloured boxes. Grey boxes indicate the loss of particular *Wnt *subfamilies and boxes with question marks indicate *Wnts *not found in some animals, but which cannot be definitively described as 'lost' because the relevant genomes have not been sequenced or require more comprehensive annotation. Duplicated *Wnts *are represented by two overlapping boxes. Note that *Wnt8 *is also called *WntD *in *Drosophila *and *Tribolium*. The phylogenetic relationships of the various animals is indicated by the tree on the left [14-16,21]. The asterisk indicates that for *WntA *an orthologue was isolated from another spider, *Cupiennius*. Note that the complete *Achaearanea Wnt6 *sequence was only identified subsequent to the phylogenetic analysis.

Our phylogenetic analyses of both sets of Wnt sequences found good support for the thirteen metazoan *Wnt *gene subfamilies and twelve protostome *Wnt *subfamilies, which corroborates the findings of several previous studies (Figure 1 and Additional files 5, 6, 7) [17-19,21]. The phylogenetic assignment of *Wnt *genes from each organism to particular subfamilies is summarised in figure 2.

Our results show that the common ancestor of the arthropods possessed members of all *Wnt *subfamilies with the exception of *Wnt3*, supporting the previous suggestion that *Wnt3 *was lost in the lineage leading to protostomes [17]. This is most strikingly evidenced by the identification of members of all the other twelve *Wnt *subfamilies in both *Daphnia *and *Platynereis *(Figures 1, 2 and Additional files 5, 6, 7).

Comparison of insects to other arthropods illustrates that the loss of *Wnt *genes appears to be more common among the insects, either through loss in the lineage leading to the insects, for example, *Wnt2 *and *Wnt4*, or losses in particular clades, for example, *Wnt16 *in holometabolous insects, *Wnt11 *in dipterans, and *WntA *in *Drosophila *(Figure 2). However there are probably also some cases of *Wnt *gene loss in non-insect arthropods, for example, *Wnt10 *may have been lost in chelicerates and myriapods (Figure 2), and we were unable to find a *Wnt2 *orthologue in *Ixodes*. In addition, we cannot exclude that there has been more extensive loss of *Wnt *genes in *Glomeris *as an alternative explanation to limitations in screening using degenerate PCR in this species.

In contrast to the patterns of *Wnt *gene loss, the presence of duplicates of *Wnt *genes appears to be less frequent. While we found duplications of both *Wnt7 *and *Wnt11 *in *Achaearanea*, no other duplications have yet been found in any other arthropod (Figure 2). Furthermore, although duplications of *Wnt5, Wnt11 *and *Wnt16 *are found in other lophotrochozoans, we found only single copies of each *Wnt *gene in *Platynereis *(Figure 2) [17].

Several *Drosophila *and *Caenorhabditis Wnt *genes have previously been described as 'orphan' genes, however, our phylogenetic analysis allowed us to assign these genes to specific subfamilies. We found strong support that *Drosophila WntD *is the *Drosophila *orthologue of *Wnt8 *[19,59] (Figures 1, 2, and Additional file 5). Moreover, while the *Caenorhabditis Wnt *genes *Cwn-1, Cwn-2 *and *lin-44 *were previously assigned to the *Wnt4, Wnt5 *and *Wnt10 *subfamilies respectively, the homology of *mom-2 *and *egl-20 *could not be determined [19]. Our analysis supports the previous assignments of *Cwn-1, Cwn-2 *and *lin-44*, and furthermore indicates that *mom-2 *and *egl-20 *are probably *Wnt9 *and *Wnt16 *orthologues respectively (Additional file 5).

### Synteny of Wnt genes

Analysis of the arrangement of *Wnt *genes on the *Daphnia *genome scaffolds revealed two syntenic clusters of these genes: *Wnt9-Wnt1-Wnt6-Wnt10 *and *Wnt5-Wnt7 *(Additional file 8). This is consistent with similar *Wnt *clusters in other metazoans, including *Nematostella*, and therefore reflects an ancient arrangement of *Wnt *genes in animals [17,60]. Indeed *Lottia gigantea *and *Daphnia *exhibit very similar organisation of these *Wnt *genes (Additional file 8). However, the precise organisation of these clusters can vary between lineages, for example, *Wnt6 *and *Wnt9 *are oriented differently in *Drosophila *and *Daphnia *(Additional file 8). Interestingly, these *Wnt *clusters may represent ancient duplications of *Wnt *genes; a hypothesis supported by the phylogenetic relationships of *wg *and *Wnt6*, and *Wnt9 *and *Wnt10 *in our study (Figure 1 and Additional file 5) and several previous studies [17-19].

### Expression of Wnt genes

To further compare the *Wnt *genes among arthropods and annelids and to investigate the possible developmental roles of these genes, we characterised the expression of these genes in *Achaearanea, Glomeris, and Platynereis *and further characterised *Wnt *genes with segmentally reiterated expression in *Tribolium *[14]. Note that the *Drosophila Wnt *gene names do not refer to homology with vertebrate *Wnt *subfamilies, but rather they were mostly named in the order they were discovered (e.g. D*Wnt2 *is actually a *Wnt7 *orthologue not a *Wnt2 *orthologue). Therefore, below, we use the gene name with respect to its vertebrate orthologue and where appropriate give the *Drosophila *name in parenthesis, with the exception of *wg *(also see Additional file 1).

### wg

**Figure 3:**
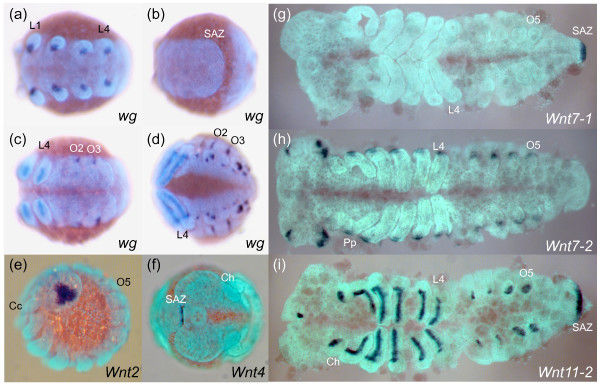
***wg, Wnt2, Wnt4, Wnt7-1, Wnt7-2 *and *Wnt11-2 *expression in *Achaearanea***. *At-wg *is expressed in the anterior ventral portion of the limb buds (a), but is not observed in ventral or dorsal regions of the prosomal segments or in the SAZ (b). Expression of *At-wg *extends along the axis of the growing legs and is observed in dorsal stripe in O2 and O3 (c) and later in O1 to O5 in groups of cells in the opisthosomal limb buds (d). *At-Wnt2 *expression is observed from stage 9 onwards in a central and lateral triangular shaped domain in the developing head lobes (e). *At-Wnt4 *is only expressed in a few cells at the very posterior of the SAZ during late embryonic development (f). Whereas *At-Wnt7-1 *is only expressed in the SAZ (g), *At-Wnt7-2 *is expressed at the base of the appendages and in a lateral anterior and posterior domain in the head lobes (h). *At-Wnt11-2 *expression appears at stage 6 in the posterior end of the embryo and persists in the SAZ throughout embryonic development (i). *At-Wnt11-2 *is also expressed in an anterior domain along the proximo-distal leg axis, the buds of the opisthosomal appendages and in the stomodeal region (i). Ch, cheliceres; Pp, pedipalps; L1 and L4, leg bearing segments; O1 to O5, opisthosomal segments; SAZ, segment addition zone. (a) to (d), ventral views of whole mounted embryos. (e), lateral view. (f), anterior view with posterior curving to the right. (g) to (i), flat mounted embryos with anterior to the left.

In *Achaearanea, wg *is expressed in stripes in the L1 and L2 segments, but only during stage 8, and such stripes are never observed in the other prosomal segments (Additional file 9: panel a). Subsequently, dots of *wg *expression associated with the developing limb buds are observed in all prosomal segments (Figure 3a, and Additional file 9: panel b). In the opisthosoma, *At-wg *is only expressed in the dorsal cells of the O2 and O3 segments (Figure 3c, d, and Additional file 9: panel b), and is not observed in the SAZ at any stage (Figure 3b). Later in development, *At-wg *expression continues in the prosomal appendages, and is also observed in opisthosomal limb buds, the labrum and the hindgut (Figure 3d, and Additional file 9: panel c).

**Figure 4:**
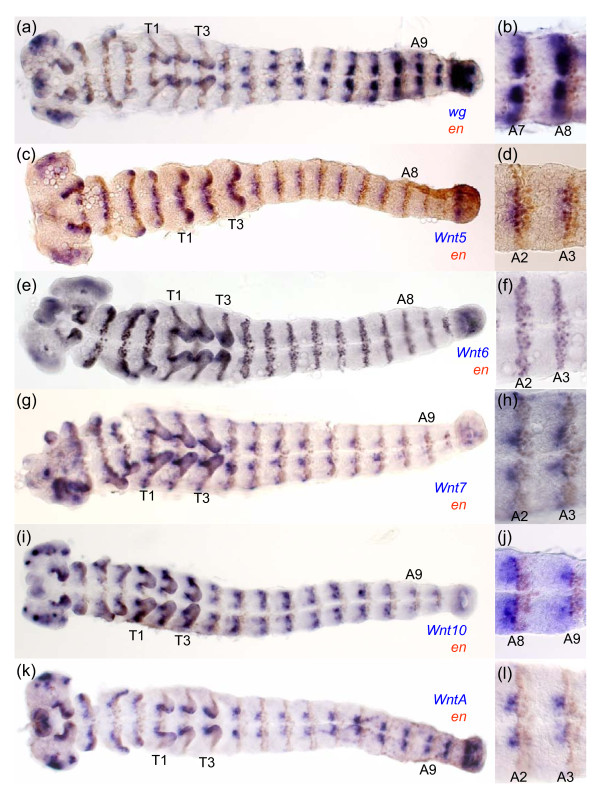
**Expression of *wg*, *Wnt5, Wnt6, Wnt7 Wnt10 and WntA *with respect to *en *in *Tribolium***. Germ band extended *Tribolium *embryos double stained for transcripts of *en *and *wg *(a), (b); *Wnt5 *(c), (d); *Wnt6 *(e), (f); *Wnt7 *(g), (h); *Wnt10 *(i), (j) and *WntA *(k), (l). High magnification images of segments are shown in (b), (d), (f), (h), (j) and (l). All embryos are shown with anterior to the left. thoracic (T1, T2) and abdominal (A2, A3, A8, A9) segments are indicated.

In contrast, in a different spider, *Cupiennius*, *wg *is expressed at the posterior of each parasegment and in the SAZ [30] consistent with classic roles in segment addition and boundary formation as described in other arthropods such as *Tribolium *(Figure 4a, b) [3,33,34,61,62]. Remarkably, this suggests that *Achaearanea *has either lost the expression and associated functions of *wg *in most segments and the SAZ or there is an additional paralogous *wg *gene in this spider not found in our PCR screen.

**Figure 5:**
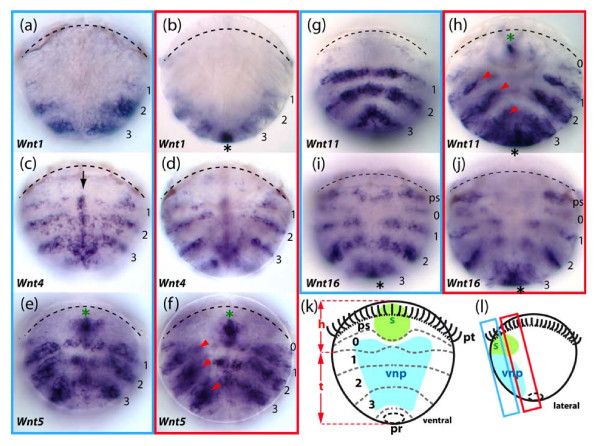
**Expression patterns of five *Wnt *genes in 48 hours post-fertilization trochophore larvae of the annelid *Platynereis***. The expression of these *Wnt *genes is observed either in reiterated ectodermal segmental stripes and/or in the pygidial/proctodeal presumptive territory. A schematic description of the trochophore larva is given in (k). pt: the prototroch, a ciliated belt used for swimming, also highlighted by a black line on larvae photographs, divides the larva into an apical episphere and a vegetal hyposphere; pr: proctodeum; s: stomodeum; ps: peristomium, a band of embryonic tissues around the forming mouth; 0: anterior-most segmental unit; 1-3: presumptive areas of the larval appendage-bearing segments. h: the future head of the worm formed by the episphere plus peristomium plus segment 0; t: the future trunk of the worm formed by larval segments 1-3 plus the pygidium. (l) shows the two approximate focal planes that are used for larvae photographs. The first and third panel columns (a), (c), (e), (g), (i) show ventral views of trochophore larvae, focusing mainly on tissues of the ventral neuroectoderm that will form the ventral nerve cord. The second and fourth panel columns (b), (d), (f), (h), (j) are frontal optical section focusing on the lateral parapodia-forming fields. The *Wnt *stripes corresponding to each presumptive larval segment are numbered 1, 2, 3. In addition, a more anterior metameric unit located just below the prototroch is numbered 0. This unit does not produce a larval segment but fuses with the head early in development. Black asterisks show expressions in the pygidial/proctodeal area. Green asterisks show expressions in the stomodeal bulb that will give rise to the mouth. The midline expression of *Pd-Wnt4 *is indicated by a black arrow. Internal *Pd-Wnt5 *and *Pd-Wnt11 *expression potentially located either in the segmental mesoderm or in ectodermal cells of the chaetal sacs are shown by red arrowheads. Additional expression of *Wnt *genes in the nascent brain are described elsewhere [84].

**Figure 6:**
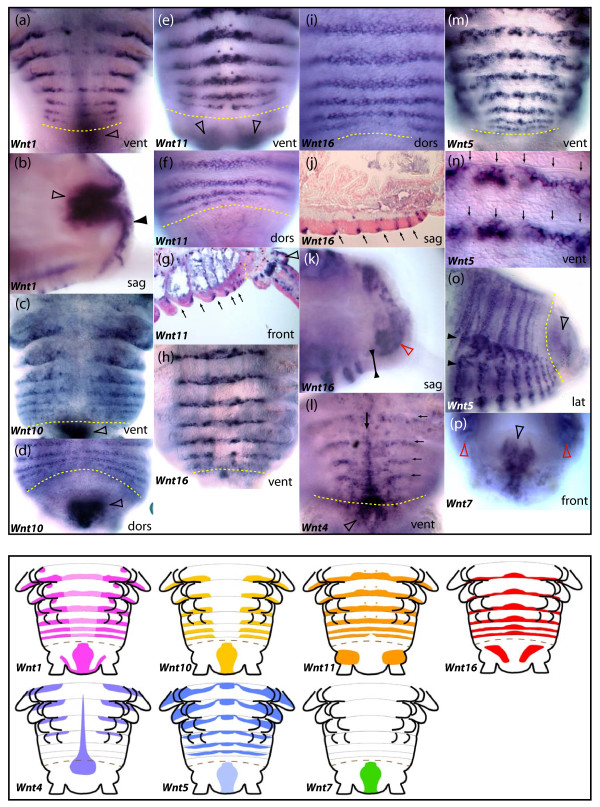
**Expression patterns of seven *Wnt *genes during posterior segment addition in the annelid *Platynereis***. All panels show series of segments produced 8 days after caudal amputation and regeneration; vent, dors, lat: ventral, dorsal and lateral views respectively. Sag and front: sagittal and frontal sections (optical or tissue) respectively. The SAZ is highlighted with a yellow dashed line in all micrographs. The pygidium is located below or right of the SAZ line, depending on the view. In (g), (j), (l), (n), arrows indicate the position of segmental grooves. (a), (b) Expression of *Wnt1 *in the posterior part of forming segments and parapodia (a), in the hindgut (hollow arrowheads) and in the ectoderm of the pygidium (black arrowhead). (c), (d) Expression of *Wnt10 *in the posterior part of forming segments and parapodia, as well as in the hindgut (hollow arrowheads). (e)-(g) Expression of *Wnt11 *in the posterior part of forming segments and parapodia, in a pair of cells of the ganglia of the ventral nerve cord (black arrowhead) and in the ectoderm at the base of the pygidial cirri (hollow arrowheads). (g)-(k) Expression of *Wnt16 *in the posterior part of forming segments but not in parapodia. (k) Expression of *Wnt16 *in the mesoderm of the pygidium (red arrowhead) but not in the ectoderm (black arrowheads). (l) Expression of *Wnt4 *in the ventral midline of forming segments (black arrow), in the ventral part of the SAZ and pygidium (hollow arrowhead) and in the anterior part of forming segments. (m)-(o) Expression of *Wnt5 *in stripes in the anterior part of forming segments and in a complex pattern in the forming parapodia. (n) Is a close up view of (m) at the level of the ventral ectoderm, showing the location of *Wnt5 *stripes posterior to the segmental grooves. (o) Shows the weaker dorsal stripes of *Wnt5 *that do not reach the dorsal midline, unlike *Wnt10*, *Wnt11 *and *Wnt16*. Black arrowheads show the forming parapodia. (p) Expression of *Wnt7 *in the hindgut (hollow black arrowhead) and broadly in the segmental mesoderm (red arrowheads). Patterns are recapitulated schematically in the lower part of the figure. All schemes are ventral views. A brown dashed line represent the SAZ. For the purpose of clarity, the expression of *Wnt5 *and *Wnt7 *in the mesoderm of forming segments has been omitted.

*wg *expression in *Platynereis *was previously described in [35]. *wg *is expressed at the posterior boundary of each segment both in the trochophore larva (Figure 5a, b) and during posterior growth (Figure 6a). During annelid posterior growth, *wg *expression is also observed in the hindgut and in the posterior-most pygidial ectoderm (Figures 6b) [35].

### Wnt2

It is likely that the *Wnt2 *subfamily was lost in the lineage leading to insects (Figure 2), and although we were unable to isolate an orthologue from *Glomeris *we assayed the expression of the *Wnt2 *genes from the spider *Achaearanea *and the annelid *Platynereis*.

In the spider *Achaearanea*, *Wnt2 *is first expressed relatively late in embryogenesis, in the ocular region of the developing cephalic lobes at stage 9, and this expression pattern persists into stage 10 (Figure 3e). We did not observe a distinct expression pattern for *Wnt2 *in *Platynereis*, possibly because of a low level of expression.

### Wnt4

It is probable that the *Wnt4 *subfamily was also lost in the lineage leading to insects, but is present in other arthropods and lophotrochozoans (Figure 2). Analysis of *Wnt4 *expression in *Achaearanea *and *Platynereis *shows it is highly divergent between chelicerates and annelids. In *Achaearanea*, *Wnt4 *expression is restricted to only few cells at the very posterior of the germ band during the later stages of embryogenesis (Figure 3f). In contrast, in *Platynereis*, *Wnt4 *is expressed in stripes in the anterior part of each segment and could therefore be involved in defining segment boundaries (Figures 5c, d, 6l). The stripes are limited to the dorsal and lateral parts of nascent embryonic and post-embryonic segments. Additionally, *Pd-Wnt4 *is expressed in a longitudinal stripe along the ventral midline in forming segments as well as the SAZ and the ventral pygidial ectoderm (Figures 5c, d, 6l).

### Wnt5

In *Tribolium*, *Wnt5 *is expressed in ventral stripes at the posterior of each parasegment and curiously in at least one row of cells in the anterior of each parasegment overlapping with *en *expression (Figure 4c, d). *Tc-Wnt5 *expression is also observed in the SAZ, distal tips of developing appendages, in the region of the labrum/stomodeum, and the ocular region of the head lobes (Figure 4c) [14].

**Figure 7:**
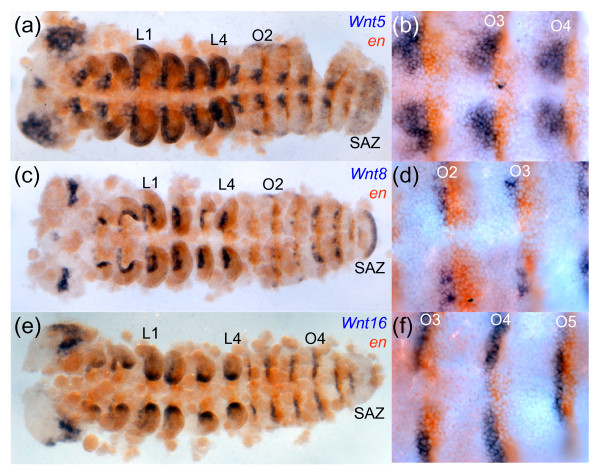
**Expression of *Wnt5, Wnt8 *and *Wnt16 *in *Achaearanea***. *At-Wnt5 *is expressed segmentally in the developing neuroectoderm directly anterior to *en *(a), (b). *At*-*Wnt5 *expression can also be detected in the head lobes, the developing labrum, the developing heart, a ring like domain in the appendages and in the SAZ (a). *At-Wnt8 *is expressed segmentally and directly anterior to *en *expression (c), (d). *At-Wnt8 *is also expressed in the SAZ, the cephalic lobes, the developing stomodeum and the appendages. *At-Wnt16 *transcripts are also found in segmental strips directly anterior to en (e), (f), as well as in the tips of the legs and in a broad domain in the developing brain. *At-Wnt16 *expression is not observed in the SAZ (e). L1 and L4, leg bearing segments; O1 to O4, opisthosomal segments; SAZ, segment addition zone. All embryos are flat mounted with anterior to the left.

In *Achaearanea*, *Wnt5 *is first expressed in a broad anterior domain (Additional file 9: panel d), and subsequently, in the cephalic lobes, throughout the SAZ, and segmentally in the developing neuroectoderm on either side the ventral midline, juxtaposed to *en *expressing cells (Figure 7a, b). *At-Wnt5 *transcripts can also be detected in a medial ring in the appendages, the labrum and the heart (Figure 7a). Similar expression patterns have been described for *Wnt5 *in *Cupiennius *[30,63].

In *Platynereis*, *Wnt5 *is also expressed in clear segmental stripes. However, in contrast to *Wnt5 *expression observed at the posterior region of parasegments in arthropods, *Pd*-*Wnt5 *is only expressed in the anterior part of segments (Figures 5e, f, 6m-o, and Additional file 10: panel f). These stripes encompass both the ectoderm and the underlying mesoderm (Figure 5f). *Pd-Wnt5 *is also expressed weakly in the hindgut during posterior growth and in a complex pattern in forming appendages.

### Wnt6

**Figure 8:**
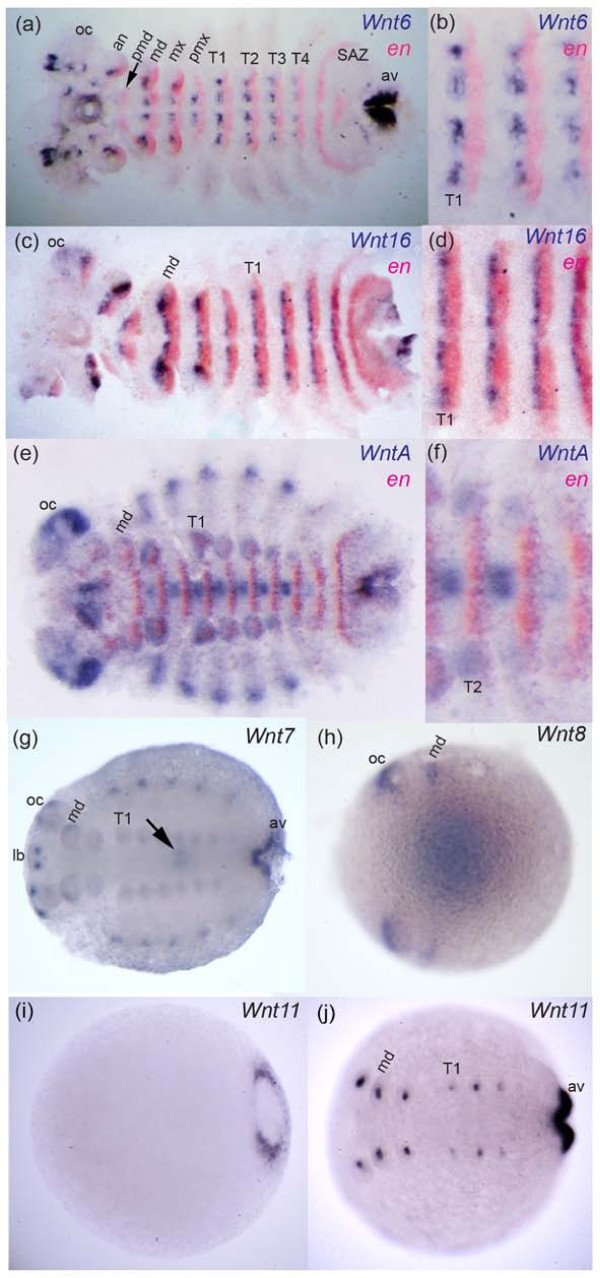
**Expression of *Wnt *genes in *Glomeris***. Stage 3 embryo double stained for *Wnt6 *and *en *(a). Higher magnification of ventral trunk segments T1 to T3 of same embryo in (a) showing abutting expression of *Wnt6 *and *en *(b). Expression of *Wnt16 *and *en *in a stage 3 embryo (c). Higher magnification of ventral trunk segments T1 to T3 of same embryo in (c) showing abutting segmental expression of *Wnt16 *and *en *(d). Expression of *WntA *and *en *in a stage 5 embryo (e). Higher magnification of ventral trunk segments T1 to T4 of same embryo in (e) showing abutting segmental expression of *WntA *and *en *(f). Stage 5 embryo stained for *Wnt7 *(g). Arrow indicates expression in the midgut. Note that *Gm-Wnt7 *expression appears to be restricted to embryos older than approximately stage 3. Expression of *Wnt8 *in a stage 0.3 embryo (h). Faint expression of *Gm-Wnt8 *at the posterior is out of focus in this picture. Coloration in the middle of the embryo is in the yolk; this artificial staining appears when over-staining *Glomeris *embryos, which was necessary to detect specific *Wnt8 *transcripts. Note that *Gm-Wnt8 *expression appears to be restricted to embryos younger than approximately stage 1. Expression of *Wnt11 *in a stage 0.3 embryo (i) and a stage 3 embryo (j). Expression of *Gm-Wnt11 *is restricted to the anal valves (av) and the growing appendages (i), (j). Expression of *Glomeris wg *is described elsewhere [32,33]. All embryos are shown with anterior to the left. Embryos in (a) to (f) are flat mounted. Abbreviations: an, antennal segment; av, anal valve; lb, labrum; md, mandibulary segment; mx, maxillary segment; OC, optic lobes; pmd, pre-mandibulary segment; pmx, post-maxillary segment; T1-T4, trunk segments one to four.

In *Tribolium*, *Wnt6 *is expressed in the developing brain, appendages, and in segmental stripes that overlap with *en *expressing cells (i.e. posterior to *wg *expression) (Figure 4e, f). *Tc-Wnt6 *is also expressed in a sub-terminal region of the SAZ (Figure 4e) [14]. In *Glomeris*, *Wnt6 *is expressed in reiterated stripes in completed segments, directly anterior to *en *expression, and at later stages is observed in dorsal patches in each segment (Figure 8a, b) similar to *Wnt6 *expression in older *Tribolium *embryos. In addition, *Gm-Wnt6 *is expressed in specific domains in the developing brain, in and at the posterior of the germ band, including expression in the anal valves (Figure 8a). Diffuse expression of *Gm-Wnt6 *is also observed in the gut later in embryogenesis (not shown).

In *Platynereis*, *Wnt6 *is expressed in the mesoderm of trochophore larvae, and in the mesodermal layer of the intestine in the growing juvenile (Additional file 10: panels a and g).

### Wnt7

In *Tribolium*, *Wnt7 *is expressed segmentally in two clusters of cells either side of the ventral midline abutting *en *expressing cells, essentially in a similar pattern to *Tc*-*wg *(Figure 4a, b, g, h) [14]. *Tc-Wnt7 *is later expressed in the dorsal of the developing limbs and in the developing brain (Figure 4g) [14].

The two *Achaearanea Wnt7 *paralogues exhibit non-overlapping expression patterns similar to subsets of *Tc-Wnt7 *expression: *At-Wnt7-1 *is expressed only in the SAZ (Figure 3g), and *At-Wnt7-2 *is expressed in the proximal dorsal region of the developing appendages and in the developing brain (Figure 3h). However, neither of the spider *Wnt7 *genes or *Glomeris Wnt7 *is expressed in a segmental pattern like *Tc-Wnt7*. *Gm-Wnt7 *is expressed in older embryos at the posterior of the germ band in the anal valves, the brain, the heart, the midgut, the labrum, the mandibles, and possibly also weakly in the other developing appendages (Figure 8g). In *Platynereis*, *Wnt7 *is expressed in the mesoderm of the larva (not shown) and later during juvenile growth (Figure 6p). *Pd-Wnt7 *is also strongly expressed in the hindgut (Figure 6p).

### Wnt8

In *Achaearanea *embryos, *Wnt8 *is expressed in the posterior most cells of the SAZ, the cephalic lobes, the developing stomodeum, the appendages, and in ectodermal stripes anterior to *en *in each segment (Figure 7c, d) [24]. Although *Wnt8 *is also expressed in the SAZ of *Tribolium *embryos, it is not expressed segmentally in this beetle [14,23].

In *Glomeris*, *Wnt8 *is expressed in two anterior domains and in the putative SAZ (albeit quite weakly), however, expression was only found in early embryos (Figure 8h) and no segmentally reiterated expression was observed.

*Platynereis Wnt8 *is expressed strongly in the future brain of the larva (Additional file 10: panel b). Faint ventral stripes are also detected in late stage trochophore larvae (Additional file 10: panel b), but no corresponding pattern is detected during posterior growth.

### Wnt9

We were unable to isolate a *Wnt9 *gene from either spider species or *Glomeris*, but this may reflect a limitation of degenerate PCR rather than a loss in these lineages because a *Wnt9 *orthologue is found in the tick *Ixodes *(Figure 2). In *Platynereis, Wnt9 *is first expressed at the posterior pole in the trochophore larva (Additional file 10: panel c). During juvenile posterior growth, it is just observed in a few cells scattered in the gut endoderm (Additional file 10: panel h).

### Wnt10

Again we were unable to isolate a *Wnt10 *gene from either *Achaearanea *or *Glomeris *and in addition no *Wnt10 *orthologue was found in the *Ixodes *gene models (Figure 2). In *Tribolium*, *Wnt10 *is expressed in a similar pattern to *wg *in the cephalic lobes, appendages and at the posterior parasegmental boundaries abutting *en *expression (Figure 4i, j) [14]. Similar to *wg/Wnt1 *expression in *Platynereis*, *Pd-Wnt10 *is expressed at the posterior boundary of each segment and in the hindgut during posterior growth (Figure 6c, d). Surprisingly, we did not observe a similar expression pattern in the trochophore larva perhaps due to probe detection limitations. Instead, two pairs of cells were stained presumably in the anterior larval mesoderm (Additional file 10: panel d).

### Wnt11

There are two *Wnt11 *genes in *Achaearanea*. While we did not detect any embryonic expression of the *Wnt11-1 *paralogue, *At-Wnt11-2 *is expressed in the SAZ starting at stage 6 (Additional file 9: panel e) and then throughout segmentation (Figure 3i). *At-Wnt11-2 *is also expressed in the developing appendages in an anterior domain along the proximo-distal axis of the prosomal appendages and in a distal domain in the buds of opisthosomal appendages (Figure 3i). In *Glomeris*, *Wnt11 *is first expressed at the posterior of the germ band (Figure 8i), and later in the anal valves, and at the tips of each appendage (Figure 8j). In the maxillae three spots of expression are also observed that resemble the expression of *wg *in *Glomeris *(Figure 8j).

In *Platynereis*, *Wnt11 *is strongly expressed in segmental stripes in the posterior part of each segment in a similar position to *wg *in the larva (Figure 5g, h) and during juvenile growth (Figure 6e-g). *Pd-Wnt11 *is also expressed in the brain (not shown), the stomodeum and the presumptive pygidium (Figure 5h). During posterior growth, it is also strongly expressed posteriorly, but in the ectoderm covering the pygidium at the base of the tentacular cirri rather than in the hindgut like other *Wnt *genes (Figure 6e).

### Wnt16

Investigation of *Wnt16 *expression in *Achaearanea*, *Glomeris *and *Platynereis *showed that in all three of these animals *Wnt16 *is expressed in segmental stripes directly anterior to *en *(Figures 5i, j, 6h-j, 7e, f, 8c, d). Thus like *wg, Wnt16 *might be involved in the generation of segmental and parasegmental boundaries in annelids and arthropods respectively (perhaps with the exception of holometabolous insects, see figure 2). In nascent segments of *Glomeris *and *Achaearanea *embryos, *Wnt16 *is observed in ventral restricted stripes (Figures 7e, f, 8c). However, in older segments *Wnt16 *is expressed in stripes either side of the ventral midline (Figures 7e, f, 8c, d). *Wnt16 *expression is also observed in the cephalic lobes and the distal tips of the appendages in the spider and millipede (Figures 7e, 8c).

In *Platynereis, Wnt16 *is expressed in segmental stripes just at the posterior border of segments (Figure 5i, j, 6h-k). Interestingly, the trochophore larvae show five stripes of *Wnt16 *of unequal strength, in addition to the three parapodia bearing larval trunk segments delineated by the other *Wnt *genes. *Pd-Wnt16 *is also expressed in the peristomium (the "ring" that carries the mouth just below the prototroch in annelids) and in a transient segmental anlage just posterior to it. Both segment-like structures fuse with the head at metamorphosis. During posterior growth, *Pd-Wnt16 *is also expressed in the pygidium mesoderm, but not in the hindgut or pygidial ectoderm like other *Wnt *genes (Figure 6k).

### WntA

Analysis of the expression of *WntA *orthologues in *Tribolium, Cupiennius *and *Platynereis *again revealed quite different patterns for this *Wnt *subfamily across protostomes. In *Tribolium, WntA *is expressed in the head lobes, appendages, SAZ and segmental stripes (Figure 4k, l) [14]. The segmental expression of *WntA *in *Tribolium *is again found anterior to *en *in a similar domain to *wg *(Figure 4l). *Glomeris WntA *is expressed in clusters of cells in the ventral neuroectoderm posterior to *en *expressing cells, at the posterior end of the germ band (weakly) and developing heart (Figure 8e, f) [33]. Expression of *WntA *is also observed in the SAZ of the spider *Cupiennius *(Additional file 9: panel g), and although we also observed expression in a distal domain in the spinnerets and a lateral spot in the cheliceres, *WntA *is not expressed segmentally in this spider (Additional file 9: panels g-i). Thus *WntA *expression is rather different between mandibulates and chelicerates.

In *Platynereis*, *Pd*-*WntA *is strongly expressed in the parapodial anlagen in larvae and during posterior growth (Additional file 10: panels e and i). *Pd-WntA *expression is later observed at the distal extremities of growing parapodia (Additional file 10: panel i). A striped expression in the mesoderm during posterior growth has probably no connection to segment formation as high magnification shows that these stripes correspond to the walls of lateral blood vessel branching from the dorsal and ventral blood vessels (Additional file 10: panel j).

## Discussion

### Ancestral composition, conservation, loss and duplication of protostome Wnt genes

It has been shown that the thirteen subfamilies of *Wnt *genes found in metazoans appeared before the evolution of bilaterians, and that thirteen and twelve subfamilies are represented in extant deuterostomes and protostomes respectively [17-22] (Figure 2). Strikingly we have now found twelve *Wnt *subfamilies in both an arthropod, the crustacean *Daphnia*, and in the annelid *Platynereis *confirming that the common ancestor of protostomes contained all *Wnt *subfamilies except *Wnt3*. Furthermore, our study, the first broad survey of *Wnt *gene diversity across arthropods, shows that the common ancestors of arthropods and ecdysozoans also contained representatives of all twelve *Wnt *subfamilies found in protostomes (Figures 1, 2, and Additional file 5).

In insects there has been extensive loss of *Wnt *genes, for example, only seven and six *Wnt *genes are found in *Drosophila *and *Acyrthosiphon *respectively [14,16]. This reflects the absence of *Wnt2 *and *Wnt4 *in all insects and lineage specific patterns of loss such as *Wnt11 *in dipterans (Figure 2). Moreover, this suggests that while the loss of *Wnt *genes has been common in insects and the nematode *Caenorhabditis*, most ecdysozoans may actually have retained a larger repertoire of these genes (Figure 2). Similarly, the leech, *Helobdella*, also appears to have lost a number of *Wnt *genes with respect to other lophotrochozoans like *Capitella *[17] and *Platynereis*. However the reasons for retention of a large repertoire of *Wnt *genes in some lineages and extensive loss in others is currently unknown.

Curiously duplications of individual *Wnt *genes (i.e. apart from those generated by whole genome duplications in deuterostomes) are rather rare (Figure 2). The reason for this could be that the concentration of individual Wnt ligands is important for the overall combinatorial output of Wnt signalling in particular tissues (see below). Indeed, in animals with *Wnt *duplications, the paralogues appear to have been subject to subfunctionalisation, as evidenced by distinct expression patterns of the *Wnt7 *and *Wnt11 *paralogues in the spider, and duplicated *Wnt *genes in lophotrochozoans [17]. Our data also support previous phylogenetic studies of *Wnt *genes suggesting ancient duplications may have given rise to clusters of *Wnt *genes, such as the *Wnt9-wg-Wnt6-Wnt10 *cluster found in *Daphnia *and other metazoans (Additional files 5 and 8).

### Combinatorial action of Wnts

**Figure 9:**
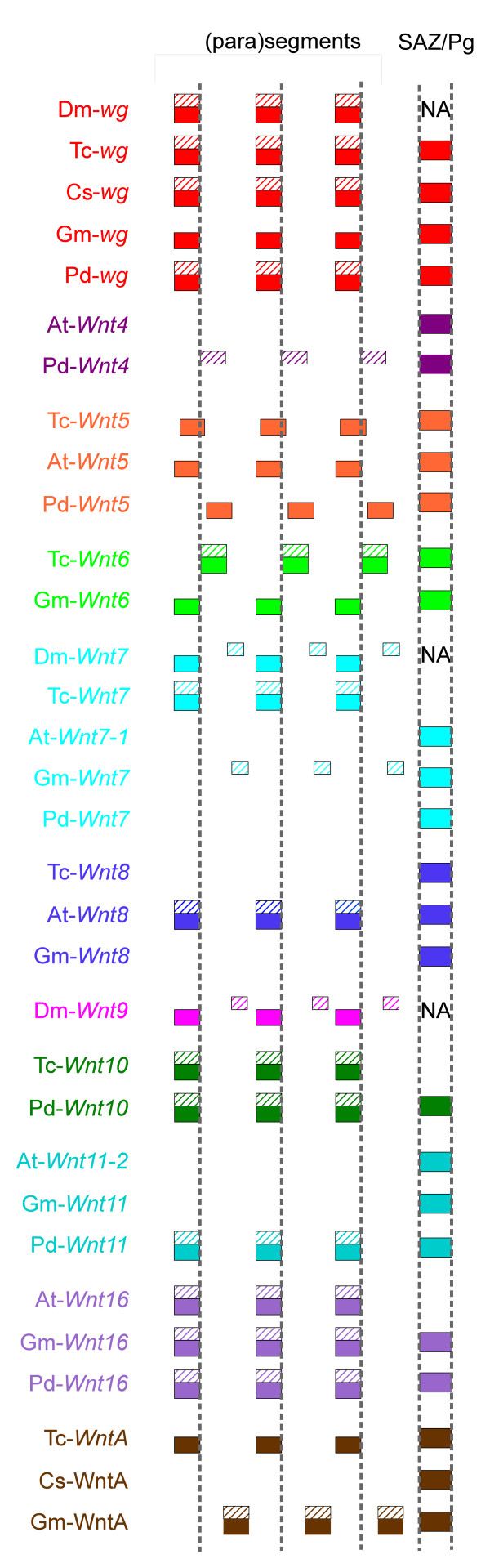
**Metameric and posterior expression of *Wnt *genes in protostomes**. Expression of *Wnt *genes is illustrated with respect to the parasegmental and segmental boundaries of arthropods and annelids respectively, and the SAZ/Pg (boundaries are represented by dashed vertical lines). Anterior is to the left. Expression in the ventral part of the segment and SAZ/Pg is shown as filled boxes and expression in the dorsal represented by hatched boxes. Note that the metameric dorsal expression of *Gm-Wnt7 *and *Gm-Wnt16 *is restricted to presumptive heart tissue. SAZ, segment addition zone; Pg, pygidium; NA, not applicable; *Dm, Drosophila melanogaster; At, Achaearanea tepidariorum; Cs, Cupiennius salei, Gm, Glomeris marginata; Pd, Platynereis dumerilii; Tc, Tribolium castaneum*.

Our present study of *Wnt *gene expression in a range of arthropods and an annelid, and previous studies in other metazoans [14,17,18,21,64,65], show that numerous *Wnt *genes are often expressed in the same cells or tissues; for example, various *Wnt *genes are expressed in the SAZ and at the same position within segments (Figure 9). Does this imply that Wnt ligands are essentially redundant? The lack of obvious phenotypic effects associated with the loss of expression of some *Wnt *genes in particular tissues suggests that they may be functionally interchangeable in certain contexts [e.g. [23,66]]. However, there are also several arguments against the general functional redundancy of these ligands. First, the fact that twelve or thirteen *Wnt *genes are retained in many animals argues against redundancy. Second, since Wnt ligands diffuse from source cells and thus can act on a range of different target cells, expression of multiple Wnts in the same cell does not necessarily mean they have the same function. Third, studies directly comparing the function of different Wnts have provided direct experimental evidence that these ligands are functionally distinct. In *Drosophila wg *and *Wnt9 (DWnt4) *have similar expression in segmental stripes, but they play different roles in ectodermal patterning [5,67], while over-expression of the other five *Drosophila Wnt *genes has no affect on cuticular patterns [64,68]. Furthermore, Llimargas and Lawrence [64] found that *wg *and *Wnt7 *(*DWnt2*) act together during *Drosophila *tracheal development, but none of the five other *Drosophila *Wnts could perform the same roles. These results, as well as those of studies in *Caenorhabditis *[e.g. [65,66]], reflect increasing evidence that Wnt signalling is more complex than simple linear signalling pathways, and that Wnt ligands expressed in similar patterns may work agonistically and antagonistically to fine tune cellular responses [42]. Indeed, it is perhaps even more realistic to think of an overall Wnt ligand landscape or code rather than the function of individual Wnts [69].

The specificity of Wnt signalling is also facilitated by the great complexity of transduction mechanisms employed [42]. Wnt ligands are capable of binding to several different receptors, including 7-pass Frizzled receptors and the receptor tyrosine kinases Ryk and Ror, which in turn are capable of activating several cross-talking cytoplasmic pathways. It has been proposed that these transduction mechanisms allow a combinatorial action of Wnt ligands in particular tissues [42]. Interestingly, however, this also opens the possibility that a given cellular response might be achieved with several different Wnt ligand combinations. Therefore, the expression of alternative Wnt combinations in a given tissue in different taxa could still generate the same intracellular signalling outcome. This may partly explain the diversification *Wnt *ligand gene expression across metazoans.

### Wnts and segmentation in protostomes

Our analysis allows the first broad comparison of *Wnt *expression patterns across the arthropods, and our characterisation of *Wnt *expression in an annelid extends this comparison to other segmented protostomes.

Arguably, one of the most interesting observations emerging from this comparison is the high proportion of *Wnt *genes expressed in segmental stripes reminiscent of segment polarity gene expression in *Drosophila *(Figure 9). In fact, no less than six *Wnt *genes show this kind of pattern in *Platynereis *and *Tribolium*, and at least five in *Glomeris *and four in *Achaearanea *(Figure 9). It is particularly striking that eleven out of twelve protostome *Wnt *genes (the exception being *Wnt2*) exhibit a striped pattern in at least one species, and no less than nine (*Wnt11 *and *Wnt4 *are the exceptions) in at least one arthropod (Figure 9). Generally, these stripes appear before the morphological appearance of segments, suggesting that some of these genes may play roles in segment formation, although others may only be involved in the ontogenesis of segmental organs rather than segmental patterning. The last common ancestor of all arthropods was undoubtedly a metameric animal, and our study suggests that a number of *Wnt *genes probably played a role in the patterning of its segments. However, only *wg *is expressed in similar stripes across all arthropod species considered here, and even the function of this gene may have changed somewhat in *Achaearanea*. Therefore, some *Wnt *genes have lost their segmental expression in some lineages, and indeed, *Wnt16 *was lost altogether in holometabolous insects (Figure 2). Conversely, some *Wnt *genes may have evolved segmental patterning functions, for example, *Wnt7 *in *Tribolium*.

Experimental approaches have also revealed differences among *Wnt *genes with respect to their role in segmental patterning. In *Drosophila*, only *wg *and *Wnt9 *(*DWnt4*) appear to regulate the establishment of the metameric pattern [5,7,64,68,70]. In *Tribolium*, while *wg *RNAi produces segmentation defects [23], RNAi against other segmentally expressed *Wnt *genes did not affect segmentation. Clearly functional data on the other *Wnt *genes in arthropods, particularly non-insect arthropods, is required to investigate the roles of these genes in segmental patterning further.

Despite differences in the expression and probably the function of *Wnt *genes across taxa, there are nevertheless some noticeable similarities: *Wnt5 *is expressed in ventral stripes in *Tribolium, Achaearanea and Platynereis*; *Wnt16 *is expressed in reiterated stripes in *Achaearanea, Glomeris *and *Platynereis*; *Wnt10 *forms stripes in *Tribolium *and *Platynereis *(Figure 9). Furthermore, with a few exceptions, segmental expression of *Wnt *genes nearly always anteriorly abuts *en *expression in arthropods (Figure 9). Together with the fact that no *Wnt *gene demarcates the segmental boundary in arthropods (with the possible exception of *Tribolium Wnt6*), this vindicates the view that parasegment boundaries are the essential organizers of segmental patterning in these animals [30,31].

It was previously proposed that the ancestral protostome was an annelid-like segmented worm, and that arthropod cuticular segmentation evolved out of frame with the ancestral segmentation [71]. This is supported by *en, wg *[35] and *hedgehog *[36] expression patterns in *Platynereis*. In this view, arthropod parasegments are an embryonic recapitulation of ancestral segmentation. In *Platynereis*, although incomplete stripes of *Wnt4 *and *Wnt5 *are found in the anterior region of segments, *wg*, *Wnt10, Wnt11 *and *Wnt16 *are all expressed in circular stripes at the posterior segmental boundaries, anterior to *en *(Figure 9), thus supporting the hypothesis that arthropod parasegments and annelid segments are homologous. It is noteworthy, however, that the analysis of the expression patterns of a complete set of *Wnt *genes in another annelid, the leech *Helobdella*, led the authors of this study to very different interpretations [17]. In the leech, the duplicated genes *Wnt11a*, *Wnt11c*, *Wnt16a *and *Wnt16b *also give striped segmental patterns but only in the late germ band stage well after the segmental pattern is already laid down, whereas in *Capitella*, *Wnt5*, *Wnt11 *and *Wnt16 *are not expressed in ectodermal stripes but rather in segmentally iterated patterns in the mesoderm [17]. Only *Capitella Wnt11 *is expressed transiently in the ectoderm of the SAZ. These discrepancies show that the actual role of *Wnt *signalling in segment formation will have to be tested in detail in non-insect arthropod and annelid models before reaching conclusions on its evolution.

### Wnt signalling and posterior development

*Drosophila *undergoes a long germ band mode of development, where all segments are formed simultaneously. In contrast most insects and other arthropods develop through variations of the short germ band mode, which is more ancestral. In the short germ band mode of development, only the anterior-most segments are initially specified and subsequently the posterior segments are added sequentially from unsegmented posterior tissue, which is often called a posterior growth zone [72,73]. However, even within arthropods, the term "growth zone" encompasses a diversity of tissue types that use different combinations of cell proliferation, movement and differentiation to generate new segments [40]. Therefore, the 'growth zone' may be more appropriately named a segment addition zone (SAZ) because sequential addition of segments is truly the key common process involved [31,40,41]. Despite differences in the process of segment addition among arthropods, it has also been argued that this is an ancestral character of bilaterians [41].

A large proportion of *Wnt *genes in *Tribolium*, *Achaearanea *and *Glomeris *embryos are expressed in the SAZ (Figure 9). The crucial role played by Wnt signalling during segment addition has been functionally demonstrated in a few arthropods. *Wnt8 *knockdown in both *Tribolium *and in *Achaearanea *resulted in a posterior truncation of the body [23,24]. A similar phenotype is obtained in *Oncopeltus *with *wg *RNAi [25], but not in *Tribolium *[23] or *Gryllus *[74], despite expression of *wg *in the SAZ of this beetle. This suggests that the respective roles of Wnt ligands during segment addition have evolved differentially among arthropod lineages, and is consistent with differences in the expression of *Wnt *genes in this region (Figure 9).

Axis truncations produced by depletions of *armadillo/β-catenin, pangolin/TCF *and *arrow/LRP5/6 *in *Gryllus, Tribolium *and *Oncopeltus *[23,25,74,75] further evidence the crucial role played by the β-catenin pathway in segment addition. Nevertheless, given the multiplicity of ligands involved, it will be important to investigate whether posterior addition of segments in arthropods is regulated by Wnt ligands through combinatorial transduction pathways [42].

Analysis of posterior expression of *Wnt genes *in the annelid *Platynereis *brings some valuable insight to understanding segment addition in protostomes. No less than six Wnt ligands are expressed in the terminal region of the annelid body, the pygidium, during axis elongation. However the annelid SAZ is located anterior to the pygidium and is represented by a thin ring of cells in which *even-skipped *and *caudal *(*cad*) are involved in regulating the synchronous mitotic cycles that produce new segments [41]. The posterior expression domains of *Platynereis wg, Wnt5, Wnt7, Wnt10, Wnt11 *and *Wnt16 *cannot completely be superimposed because they cover the hindgut, the external pygidial ectoderm, and the pygidium mesoderm. However none of these *Platynereis Wnt *genes is actually expressed in the SAZ *sensu stricto*, suggesting that they act from a posterior signalling centre located in the mitotically quiescent pygidium and separate from the proliferating cells that are the source of the new segments [41].

In the short germ band arthropods considered in this work, the detailed organization of the SAZ is largely unknown and therefore it is not known if there is a separate segment founder cell zone and putative signalling centre that differentially express *Wnt *genes. Clearly some arthropod *Wnt *genes are expressed in the proctodeum towards the end of embryogenesis, and thus in a location homologous to the annelid hindgut. Interestingly, the posterior expression of *wg *in an arthropod with anamorphic development (segments are added during larval development), the crustacean *Triops*, shows two separate domains: a complete ring near or in the SAZ and the hindgut [62].

It has been shown that knockdown of the posteriorly expressed *Wnt8 *in a spider perturbs the posterior expression of *cad *and Delta/Notch pathway components [24]. Given similar observations in several vertebrates [76-79], a *Wnt *signalling centre acting upstream of *cad *and the Delta/Notch pathway may have regulated posterior development in the last common ancestor of bilaterian animals (*Urbilateria*) [80,81]. This interpretation is further strengthened by the arthropod expression data in our study. Moreover, we also found evidence for a posterior Wnt signalling centre in a distantly related protostome group, the annelids, in which *cad *and Delta/Notch are also involved in posterior addition [41,82,83]. However, the evolution of posterior Wnt signalling has likely been complex in bilaterians, for example, *Wnt8 *is not expressed at this location in annelids and therefore its role must be played by one or several other *Wnts *ligands in these animals.

## Conclusions

We have found evidence that combinations of many *Wnt *genes probably regulate segment addition and patterning across protostomes. However further functional studies in a range of protostomes are required to investigate the precise roles of these ligands during these important developmental processes. As well as giving greater insights into the complexities of Wnt signalling, such analyses will also allow questions regarding the evolution of segmentation [37,38] to be addressed further.

## Authors' contributions

Experiments were conceived by APM, GB, RJ, WGMD, FP, SJB and RB. Experiments were performed by RJ, MLG, GB, MP, FP, RB, ES, CH and APM. Analysis of the data was carried out by all authors. The paper was written by APM, GB, WGMD, RJ, CK and MV. All authors contributed to revising early versions of the manuscript and read the final version.

## Supplementary Material

Additional file 1 **Table of species and *Wnt *genes used in this study**. Click here for file

Additional file 2 **Table of degenerate primer sequences**. Click here for file

Additional file 3 **Alignment of 93 Wnt amino acid sequences from *Achaearanea, Acyrthosiphon***. *Cupiennius, Daphnia, Drosophila, Glomeris, Homo, Ixodes, Platynereis *and *Tribolium*. Click here for file

Additional file 4 **Alignment of 147 Wnt amino acid sequences from *Achaearanea, Acyrthosiphon, Caenorhabditis, Capitella, Cupiennius, Daphnia, Drosophila, Glomeris, Helobdella, Homo, Ixodes, Lottia, Nematostella, Platynereis *and *Tribolium***. Click here for file

Additional file 5 **Maximum likelihood tree of metazoan Wnt amino acid sequences from set 2**. Bootstrap values from Maximum likelihood analysis are given on branches. Wnt amino acid sequences were used from the following species: *Achaearanea tepidariorum (At), Acyrthosiphon pisum (Ap), Caenorhabditis elegans (Ce), Capitella teleta (Ct), Cupiennius salei (Cs), Daphnia pulex (Dp), Drosophila melanogaster (Dm), Glomeris marginata (Gm), Helobdella robusta (Hr), Homo sapiens (Hs), Ixodes scapularis (Is), Lottia gigantea (Lg), Nematostella vectensis (Nv), Platynereis dumerilii (Pd) *and *Tribolium castaneum (Tc)*. Click here for file

Additional file 6 **Maximum likelihood tree of Wnt amino acid sequences from set 1**. Bootstrap values are given on branches. Wnt amino acid sequences were used from the following species: *Achaearanea tepidariorum (At), Acyrthosiphon pisum (Ap), Cupiennius salei (Cs), Daphnia pulex (Dp), Drosophila melanogaster (Dm), Glomeris marginata (Gm), Homo sapiens (Hs), Ixodes scapularis (Is), Platynereis dumerilii (Pd) *and *Tribolium castaneum (Tc)*. Click here for file

Additional file 7 **Bayesian tree of Wnt amino acid sequences from set 1**. Posterior probabilities are given on branches. Wnt amino acid sequences were used from the following species: *Achaearanea tepidariorum (At), Acyrthosiphon pisum (Ap), Cupiennius salei (Cs), Daphnia pulex (Dp), Drosophila melanogaster (Dm), Glomeris marginata (Gm), Homo sapiens (Hs), Ixodes scapularis (Is), Platynereis dumerilii (Pd) *and *Tribolium castaneum (Tc)*. Click here for file

Additional file 8 **Synteny of *Wnt *genes in metazoans**. Position and orientation of syntenic *Wnt *genes in *Drosophila melanogaster*, *Tribolium castaneum*, *Apis mellifera*, *Daphnia pulex*, *Lottia gigantea *and *Nematostella vectensis*. The sizes of the clusters are not drawn to scale. Note that *Wnt5 *and *Wnt7 *gene are found in *Drosophila, Tribolium *and *Apis *but are not clustered in these species. Click here for file

Additional file 9 ***wg, Wnt5, Wnt11 and Wnt16 *expression in *Achaearanea*, and *WntA *expression in *Cupiennius***. *At-wg *expression is first detected at stripes in L1 and L2 (a). By stage 9, *At-wg *is expressed in anteroventral regions of the prosomal limb buds and dots in the dorsal of O2 and O3, but no expression is seen in the other opisthosomal segments or in the SAZ (b). Later at stage 10, *At-wg *is expressed as stripes in O2 and O3 and expression is also observed in the labrum and the hindgut (c). *At-Wnt5 *expression is first observed in an anterior stripe at stage 5 that broadens during stage 6 (d). *At-Wnt11-2 *is first expressed at the posterior pole of the embryo during stage 6 (e). Similar to *At-Wnt5, At-Wnt16 *expression is observed as a broad anterior stripe at stage 6 (f). Strong expression of *Cs-WntA *is visible in the SAZ (g), (g'). Weaker expression of *Cs-WntA *is also detectable at the distal ends of the spinnerets (h), (h') and in two small spots in the cheliceres (i), (i') indicated by arrows. Lateral views are shown in (a), (b), (g) and (g'), ventral views with posterior wrapping to the right in (c), (d) and (f), posterior view with dorsal up in (e), ventral views with posterior to the right in (h) and (h'), and anterior views with posterior to the right in (i) and (i'). Brightfield and DAPI counterstained images of the same embryos are shown in (g), (h), (i) and (g'), (h'), (i') respectively. Ch, cheliceres; Lb, labrum; L1 and L4, leg bearing segments; O1 to O5, opisthosomal segments; SAZ, segment addition zone. Click here for file

Additional file 10 **Additional expression patterns of *Wnt *ligand genes in the annelid *Platynereis***. (a)-(e) ventral views of 48 hpf trochophores. The black dashed line is the prototroch. Red arrowheads: Broad *Pd-Wnt6 *expression in the mesodermal bands (a) and in few cells of the anterior mesoderm for *Pd-Wnt10 *(d); Black hollow arrowhead: *Pd-Wnt9 *expression in the proctodeum; Black asterisk: *Pd-WntA *expression in the stomodeum; pX: *Pd-WntA *expression in the setal sacs. (f)-(j) details of *Wnt *gene expression during posterior growth. (f) Frontal optical section of a 7-day regenerate; Red arrowhead: striped *Pd*-*Wnt5 *expression in the mesoderm and ectoderm of forming segments. (g) Ventral view of a 7-day regenerate showing the mesodermal expression of *Pd-Wnt6*. (h) Transverse section in a nascent segment of a 7-day regenerate, showing isolated cells in the gut expressing *Pd-Wnt9*; g: gut lumen; end: gut endoderm; gm: gut mesoderm; dlm: dorsal longitudinal muscles; vlm: ventral longitudinal muscles; vnc: ventral nerve cord; p: parapodia. (i) Ventral view of a 7-day regenerate showing parapodial expression of *Pd-WntA*. (j) Close up dorsal view of nascent segments in a 7-day regenerate, showing *Pd-WntA *expression in the walls of lateral vessels (red arrowheads) branching from the dorsal longitudinal vessel (dlv). The yellow dashed line in (e), (g), (i) is the approximate position of the SAZ. Click here for file
