# Supplementary material for: Conservation, loss, and redeployment of Wnt ligands in protostomes: implications for understanding the evolution of segment formation
Source: BMC Evol Biol. 2010 Dec 1;10:374. doi: 10.1186/1471-2148-10-374 (PMC3003278; doi:10.1186/1471-2148-10-374)
Supplement: Additional file 1 — Table of species and Wnt genes used in this study. [file 1471-2148-10-374-S1.DOC]

**Additional file 1**. Table of species and *Wnt* genes used in this study.

Species Gene Accession No.* Reference

*Achaearanea tepidariorum wg* AB167808.1 Akiyama-Oda, Oda unpublished

*Achaearanea tepidariorum Wnt2* AB167813.1 Akiyama-Oda, Oda unpublished

*Achaearanea tepidariorum Wnt4* HQ650544 This work

*Achaearanea tepidariorum Wnt5* AB167810.1 Akiyama-Oda, Oda unpublished

*Achaearanea tepidariorum Wnt6* HQ650545 This work

*Achaearanea tepidariorum Wnt7-1* AB167809.1 Akiyama-Oda, Oda unpublished

*Achaearanea tepidariorum Wnt7-2* AB167811.1 Akiyama-Oda, Oda unpublished

*Achaearanea tepidariorum Wnt8* FJ013049.1 [24]

*Achaearanea tepidariorum Wnt11-1* HQ650546 This work

*Achaearanea tepidariorum Wnt11-2* HQ650547 This work

*Achaearanea tepidariorum* *Wnt16*  AB167812.1 Akiyama-Oda, Oda unpublished

*Acyrthosiphon pisum wg* XM_001945260.1 [16]

*Acyrthosiphon pisum Wnt5* XM_001949632.1 [16]

*Acyrthosiphon pisum Wnt7* XM_001948506.1 [16]

*Acyrthosiphon pisum Wnt11* XM_001944602.1 [16]

*Acyrthosiphon pisum Wnt16* XM_001946899.1 [16]

*Acyrthosiphon pisum WntA* XM_001947365.1 [16]

*Caenorhabditis elegans Cwn-1*  NM_001027076 [12]

*Caenorhabditis elegans Cwn-2* NM_069421 [12]

*Caenorhabditis elegans lin44* CEU22179 [6]

*Caenorhabditis elegans mom-2* NM_072753 [13]

*Caenorhabditis elegans egl-20*  AF103732 [8]

*Cupiennius salei WntA* HQ650548 This work

*Daphnia pulex wg* 290640 This work

*Daphnia pulex Wnt2* 43478 This work

*Daphnia pulex Wnt4* 59164 This work

*Daphnia pulex Wnt5* 64675 This work

*Daphnia pulex Wnt6* 290641 This work

*Daphnia pulex Wnt7* 64642 This work

*Daphnia pulex Wnt8* 315869 This work

*Daphnia pulex Wnt9* 290643 This work

*Daphnia pulex Wnt10* 290646 This work

*Daphnia pulex Wnt11* 290647 This work

*Daphnia pulex Wnt16* 48424 This work

*Daphnia pulex WntA* 290648 This work

*Drosophila melanogaster wg* NM_078778.3 [3]

*Drosophila melanogaster Wnt5 (Dwnt3)* NM_057576.3 [11]

*Drosophila melanogaster Wnt6*  NM_135264.2 [7]

*Drosophila melanogaster Wnt7 (DWnt2)* NM_057462.3 [11]

*Drosophila melanogaster Wnt8 (WntD)* NM_142015.2 [4, 85]

*Drosophila melanogaster Wnt9 (DWnt4)* NM_057624.2 [5]

*Drosophila melanogaster Wnt10* NM_135265.2 [7]

*Glomeris marginata wg* AJ616907.1 [33]

*Glomeris marginata Wnt6* FN796441 This work

*Glomeris marginata Wnt7* FN796442 This work

*Glomeris marginata Wnt8* FN796443 This work

*Glomeris marginata Wnt11* FN796444 This work

*Glomeris marginata Wnt16* AJ616909.1 [33]

*Glomeris marginata WntA* AJ616908.1 [33]

*Homo sapiens Wnt1* X03072 [86]

*Homo sapiens Wnt2a* NM_003391 [87]

*Homo sapiens Wnt2b* NM_004185 [88]

*Homo sapiens Wnt3* NM_030753 [89]

*Homo sapiens Wnt3a* NM_033131 [90]

*Homo sapiens Wnt4* NM_030761.4 [91]

*Homo sapiens Wnt5a* NM_003392 [92]

*Homo sapiens Wnt5b* NM_030775.2 [93]

*Homo sapiens Wnt6* NM_006522 [94]

*Homo sapiens Wnt7a* NM_004625 [95]

*Homo sapiens Wnt7b* NM_058238 [96]

*Homo sapiens Wnt8a* NM_058244 [97]

*Homo sapiens Wnt8b* NM_003393 [98]

*Homo sapiens Wnt9a* NM_003395 [99]

*Homo sapiens Wnt9b* NM_003396 [99]

*Homo sapiens Wnt10a* NM_025216 [100]

*Homo sapiens Wnt10b* NM_003394 [101]

*Homo sapiens Wnt11* NM_004626 [102]

*Homo sapiens Wnt16* NM_057168 [103]

*Ixodes scapularis* *wg* XM_002407148.1 [44]

*Ixodes scapularis Wnt4* XM_002435998.1 [44]

*Ixodes scapularis Wnt5* XM_002403186.1 [44]

*Ixodes scapularis Wnt6* XM_002403232.1 [44]

*Ixodes scapularis Wnt7* XM_002401762.1 [44]

*Ixodes scapularis Wnt8* EW836726.1

*Ixodes scapularis Wnt9* XM_002407147.1 [44]

*Ixodes scapularis Wnt11* XM_002434143.1 [44]

*Ixodes scapularis Wnt16* XM_002401743.1 [44]

*Ixodes scapularis WntA* XM_002402476.1 [44]

*Nematostella vectensis wg* AY530300.1 [21]

*Nematostella vectensis Wnt2* AY725201.1 [21]

*Nematostella vectensis Wnt3* DQ492689.1 [22]

*Nematostella vectensis Wnt4* AY687348.1 [21]

*Nematostella vectensis Wnt5* AY725202.1 [21]

*Nematostella vectensis Wnt6* AY725203.1 [21]

*Nematostella vectensis Wnt7* AY687350.1 [21]

*Nematostella vectensis Wnt8* AY792510.1 [21]

*Nematostella vectensis Wnt8b* AY725205.1 [21]

*Nematostella vectensis Wnt10* AY530301.1 [21]

*Nematostella vectensis Wnt11* AY687349 [21]

*Nematostella vectensis Wnt16* DQ492688.1 [22]

*Nematostella vectensis WntA* AY534532.1 [21]

*Platynereis dumeilii wg* AJ491796.1 [19]

*Platynereis dumeilii Wnt2* AJ491797.1 [19]

*Platynereis dumeilii Wnt4* AJ491798.1 [19]

*Platynereis dumeilii Wnt5* HM179275.1 [36] and this work

*Platynereis dumeilii Wnt6* HQ413681 This work

*Platynereis dumeilii Wnt7* HQ413682 This work

*Platynereis dumeilii Wnt8* GU169426.1 [84] and this work

*Platynereis dumeilii Wnt9* AJ491799.1 [19]

*Platynereis dumeilii Wnt10* AJ491800.1 [19]

*Platynereis dumeilii Wnt11* HM179276.1 [36] and this work

*Platynereis dumeilii Wnt16* HQ413683 This work

*Platynereis dumeilii WntA* AJ491801.1 [19]

*Tribolium castaneum wg* NM_001114350.1 [14]

*Tribolium castaneum Wnt5* XM_969591.1 [14]

*Tribolium castaneum Wnt6* NM_001170666.1 [14]

*Tribolium castaneum Wnt7* XM_968066.1 [14]

*Tribolium castaneum Wnt8* XM_966346.1 [14]

*Tribolium castaneum Wnt9* XM_962805.1 [14]

*Tribolium castaneum Wnt10* XM_963117.2 [14]

*Tribolium castaneum Wnt11* XM_964168.1 [14]

*Tribolium castaneum WntA* XM_967800.1 [14]

* *Daphnia pulex*, wfleabase: http://wfleabase.org/

*Accession numbers for Capitella teleta*, *Helobdella robusta* and *Lottia gigantea* Wnt genes from the genome website of each species are given in Cho et al., 2010.
