# Supplementary material for: Conservation, loss, and redeployment of Wnt ligands in protostomes: implications for understanding the evolution of segment formation
Source: BMC Evol Biol. 2010 Dec 1;10:374. doi: 10.1186/1471-2148-10-374 (PMC3003278; doi:10.1186/1471-2148-10-374)
Supplement: Additional file 2 — Table of degenerate primer sequences. [file 1471-2148-10-374-S2.DOC]

**Additional file 2**. Table of degenerate primer sequences*.

Name Sequence Reference

wg-fw-1 ATHGARWSNTGYACNTGYGAYTA [30]

wg-bw ACYTWRCARCACCANTGRAANGTRCA [30]

wg-fw-2 TGGGARTGGGGNGGNTGYWSNGA [30]

wnt8F1 TGGGAYMGNTGGAAYTGYCC [24]

wnt8F2 TGGGGNGGNTGYWSNGA [24]

wnt8R1 NAYNCCRTGRCAYTTRCA [24]

wnt8R2 RTCNSWRCANCCNCCCCA [24]

wntF3 TGGGGNGGNTGYGCNGA This work

wntRk RCARCACCARTGRAAYTTRCA This work

wntF6 RARTGYAARTGYCAYGG This work

wntF7 TGYSARHTNAARACNTGYTGG This work

wntR7 RTRNCCNCKNCCRCARCA This work

Wnt7-W1 GNGARGCNGCNTTYACNTAYGC This work

Wnt7-W2 GARGCNGCNTTYACNTAYGCNAT This work

Wnt7-W3 GGNTGGAARTGGGGNGGNTG This work

Wnt7-W4 YTNATGAAYYTNCAYAAYAA This work

Wnt7-C1 TGNGTRTTRTANCCNCKNCCRCA This work

Wnt7-C2 GTRTTRTANCCNCKNCCRCARCA This work

WNT10C1 GCNTWYGCNATHWSNGCNGCNGG This work

WNT10C2 TWYGCNATHWSNGCNGCNGGNGT This work

WNT16W1 CARTTYMGNCAYGANMGNTGGAA This work

WNT16W2 TTYMGNCAYGANMGNTGGAAYTG This work

WNT16W1 CANCKNACRTARCARCACCA This work

WNT16W2 TARCARCACCANNNRAAYTTRCA This work

Wnt16C0 YTCCATNGTYTCRCANGTYTTRCA This work

Wnt16C3 RCARTCNGTNARRTTNCCNGC This work

Wnt16C4 GCNSWNGTDATNGCRTADATRAANGC This work

*Combinations of primers and conditions used in PCR are available upon request
