# Supplementary figures and images for: Conservation, loss, and redeployment of Wnt ligands in protostomes: implications for understanding the evolution of segment formation
Source: BMC Evol Biol. 2010 Dec 1;10:374. doi: 10.1186/1471-2148-10-374 (PMC3003278; doi:10.1186/1471-2148-10-374)

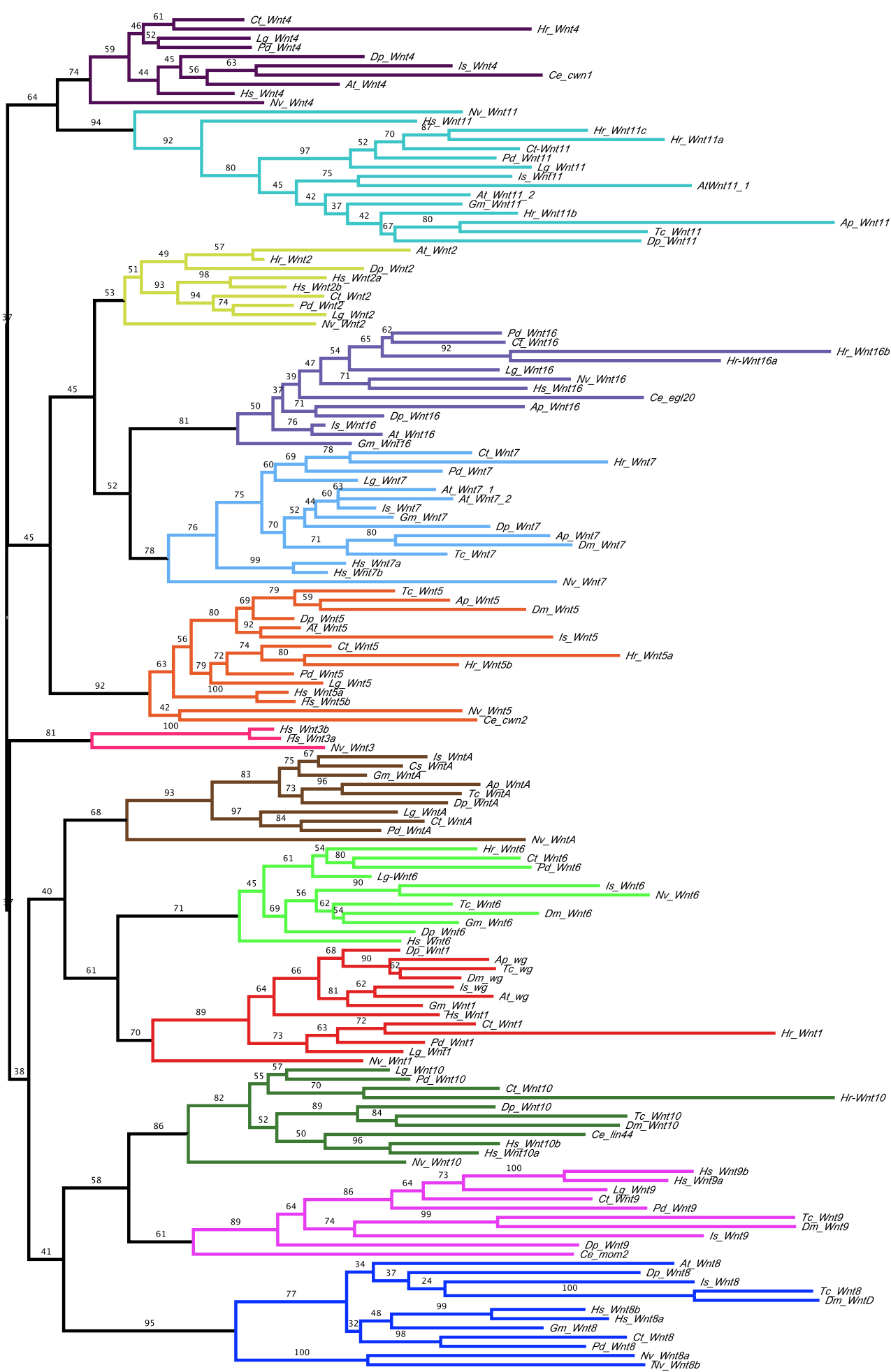

Supplement: Additional file 5 — Maximum likelihood tree of metazoan Wnt amino acid sequences from set 2. Bootstrap values from Maximum likelihood analysis are given on branches. Wnt amino acid sequences were used from the following species: Achaearanea tepidariorum (At), Acyrthosiphon pisum (Ap), Caenorhabditis elegans (Ce), Capitella teleta (Ct), Cupiennius salei (Cs), Daphnia pulex (Dp), Drosophila melanogaster (Dm), Glomeris marginata (Gm), Helobdella robusta (Hr), Homo sapiens (Hs), Ixodes scapularis (Is), Lottia gigantea (Lg), Nematostella vectensis (Nv), Platynereis dumerilii (Pd) and Tribolium castaneum (Tc). [file 1471-2148-10-374-S5.PDF]

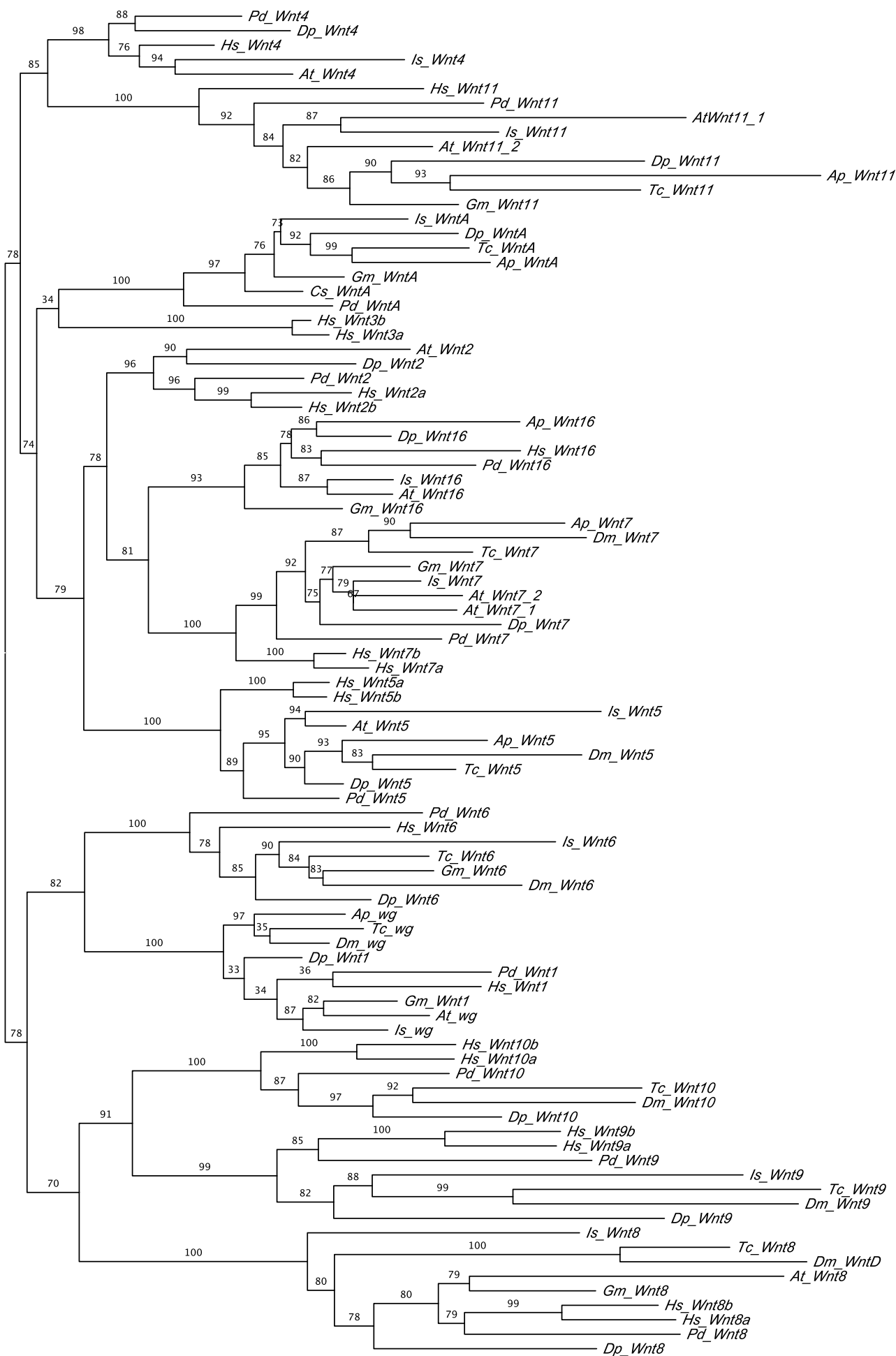

0.1

Supplement: Additional file 6 — Maximum likelihood tree of Wnt amino acid sequences from set 1. Bootstrap values are given on branches. Wnt amino acid sequences were used from the following species: Achaearanea tepidariorum (At), Acyrthosiphon pisum (Ap), Cupiennius salei (Cs), Daphnia pulex (Dp), Drosophila melanogaster (Dm), Glomeris marginata (Gm), Homo sapiens (Hs), Ixodes scapularis (Is), Platynereis dumerilii (Pd) and Tribolium castaneum (Tc). [file 1471-2148-10-374-S6.PDF]

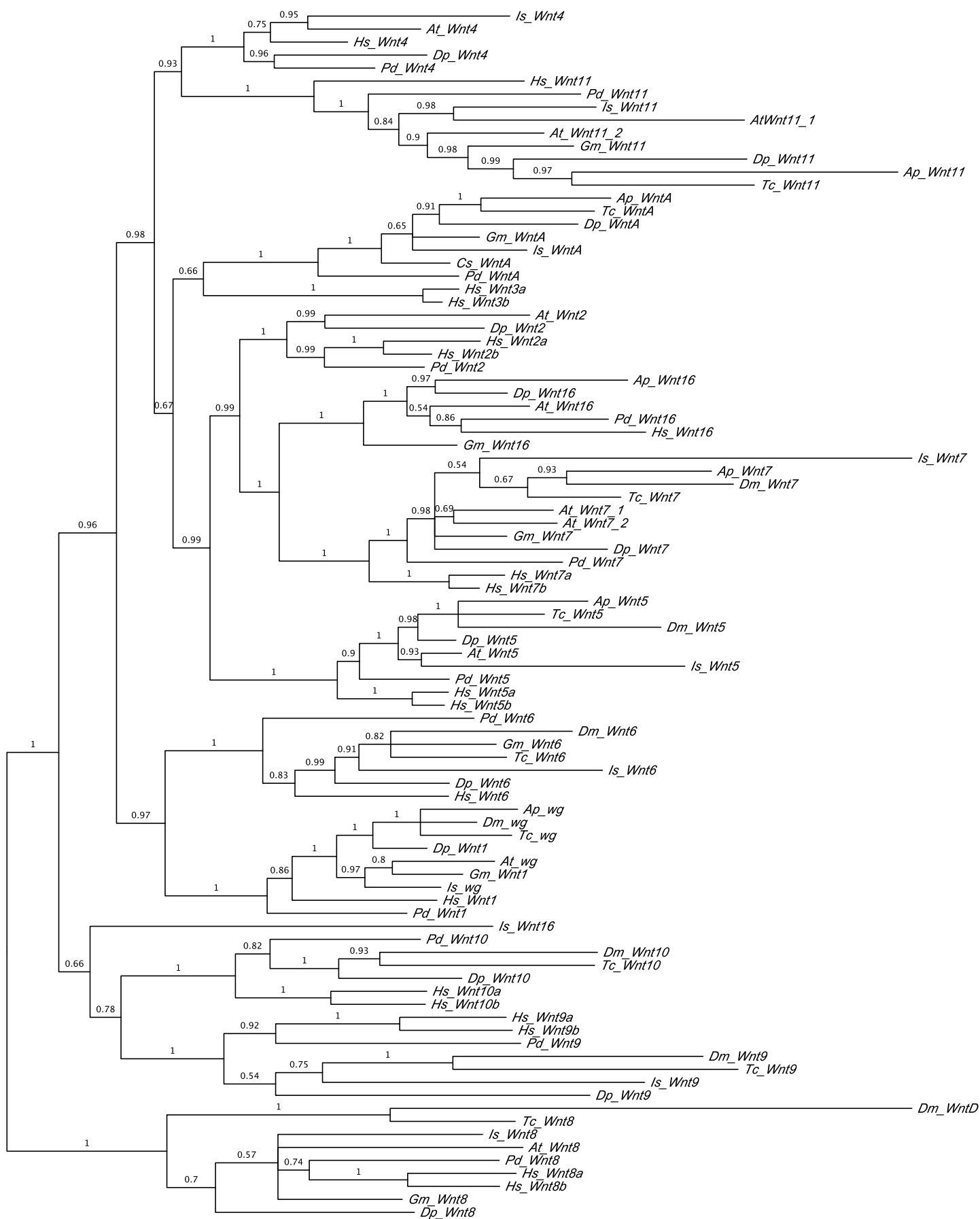

0.1

Supplement: Additional file 7 — Bayesian tree of Wnt amino acid sequences from set 1. Posterior probabilities are given on branches. Wnt amino acid sequences were used from the following species: Achaearanea tepidariorum (At), Acyrthosiphon pisum (Ap), Cupiennius salei (Cs), Daphnia pulex (Dp), Drosophila melanogaster (Dm), Glomeris marginata (Gm), Homo sapiens (Hs), Ixodes scapularis (Is), Platynereis dumerilii (Pd) and Tribolium castaneum (Tc). [file 1471-2148-10-374-S7.PDF]

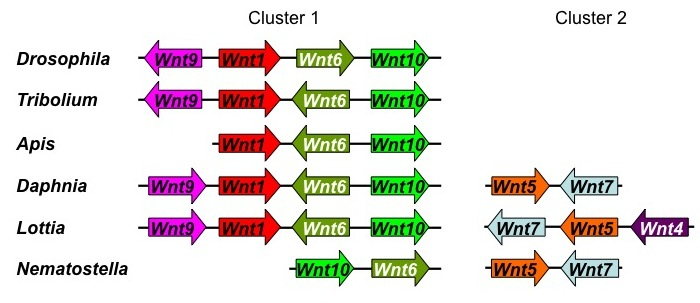

Supplement: Additional file 8 — Synteny of Wnt genes in metazoans. Position and orientation of syntenic Wnt genes in Drosophila melanogaster, Tribolium castaneum, Apis mellifera, Daphnia pulex, Lottia gigantea and Nematostella vectensis. The sizes of the clusters are not drawn to scale. Note that Wnt5 and Wnt7 gene are found in Drosophila, Tribolium and Apis but are not clustered in these species. [file 1471-2148-10-374-S8.JPEG]

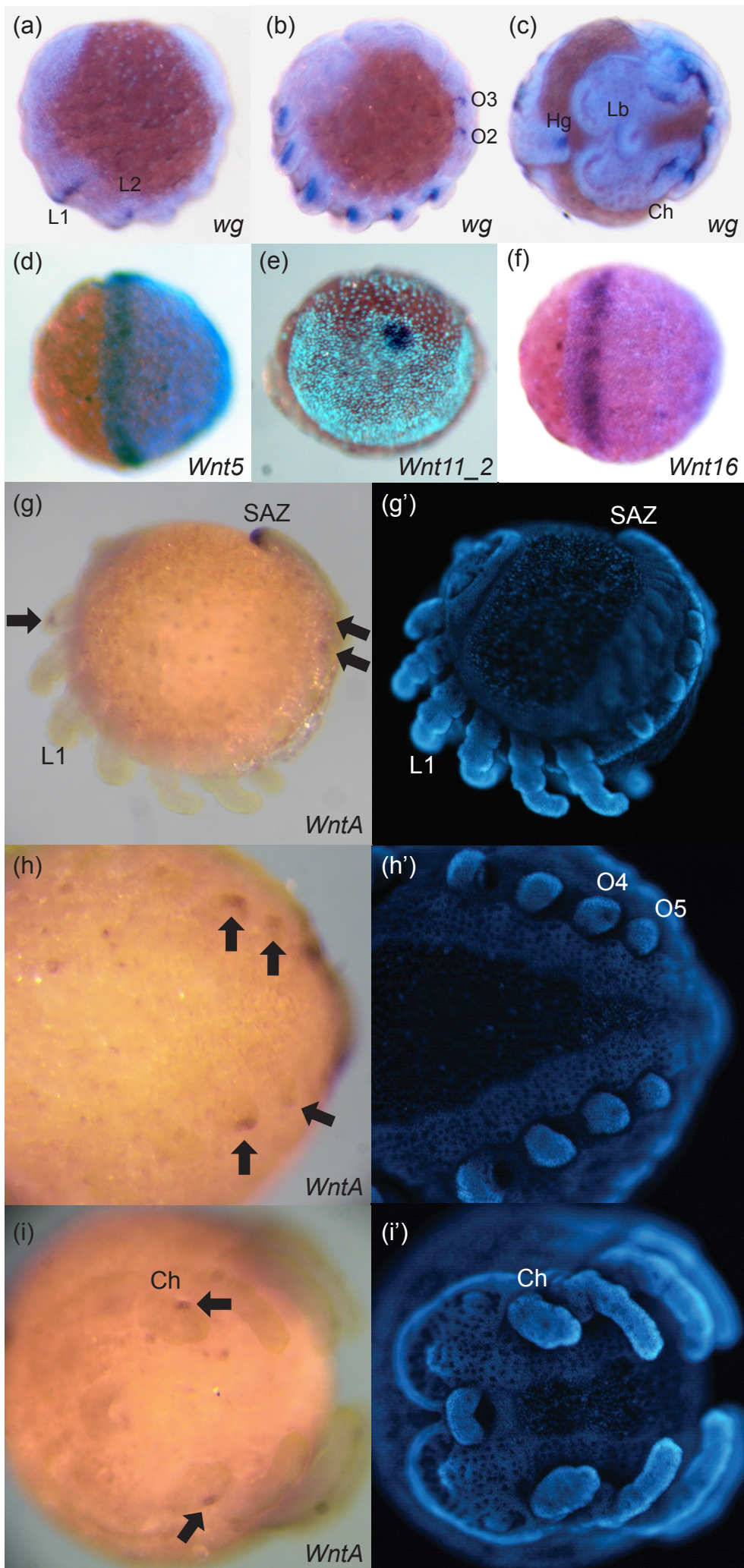

Supplement: Additional file 9 — wg, Wnt5, Wnt11 and Wnt16 expression in Achaearanea, and WntA expression in Cupiennius. At-wg expression is first detected at stripes in L1 and L2 (a). By stage 9, At-wg is expressed in anteroventral regions of the prosomal limb buds and dots in the dorsal of O2 and O3, but no expression is seen in the other opisthosomal segments or in the SAZ (b). Later at stage 10, At-wg is expressed as stripes in O2 and O3 and expression is also observed in the labrum and the hindgut (c). At-Wnt5 expression is first observed in an anterior stripe at stage 5 that broadens during stage 6 (d). At-Wnt11-2 is first expressed at the posterior pole of the embryo during stage 6 (e). Similar to At-Wnt5, At-Wnt16 expression is observed as a broad anterior stripe at stage 6 (f). Strong expression of Cs-WntA is visible in the SAZ (g), (g'). Weaker expression of Cs-WntA is also detectable at the distal ends of the spinnerets (h), (h') and in two small spots in the cheliceres (i), (i') indicated by arrows. Lateral views are shown in (a), (b), (g) and (g'), ventral views with posterior wrapping to the right in (c), (d) and (f), posterior view with dorsal up in (e), ventral views with posterior to the right in (h) and (h'), and anterior views with posterior to the right in (i) and (i'). Brightfield and DAPI counterstained images of the same embryos are shown in (g), (h), (i) and (g'), (h'), (i') respectively. Ch, cheliceres; Lb, labrum; L1 and L4, leg bearing segments; O1 to O5, opisthosomal segments; SAZ, segment addition zone. [file 1471-2148-10-374-S9.PDF]

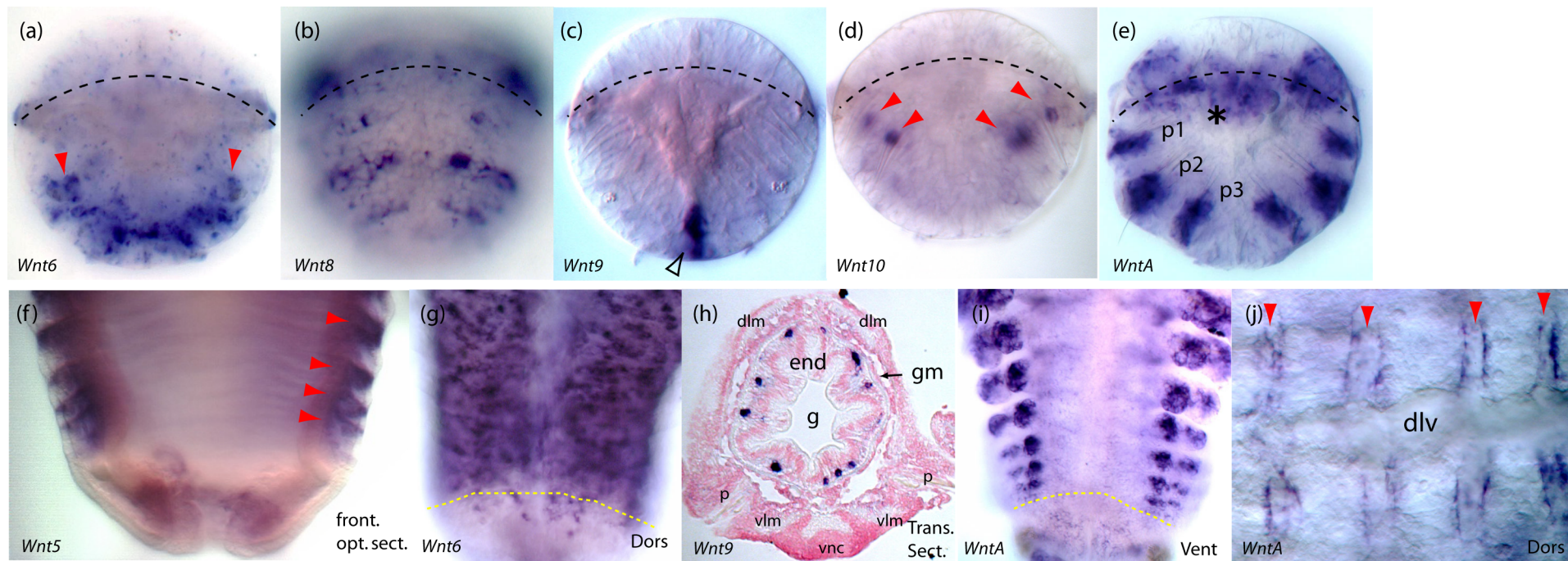

Supplement: Additional file 10 — Additional expression patterns of Wnt ligand genes in the annelid Platynereis. (a)-(e) ventral views of 48 hpf trochophores. The black dashed line is the prototroch. Red arrowheads: Broad Pd-Wnt6 expression in the mesodermal bands (a) and in few cells of the anterior mesoderm for Pd-Wnt10 (d); Black hollow arrowhead: Pd-Wnt9 expression in the proctodeum; Black asterisk: Pd-WntA expression in the stomodeum; pX: Pd-WntA expression in the setal sacs. (f)-(j) details of Wnt gene expression during posterior growth. (f) Frontal optical section of a 7-day regenerate; Red arrowhead: striped Pd-Wnt5 expression in the mesoderm and ectoderm of forming segments. (g) Ventral view of a 7-day regenerate showing the mesodermal expression of Pd-Wnt6. (h) Transverse section in a nascent segment of a 7-day regenerate, showing isolated cells in the gut expressing Pd-Wnt9; g: gut lumen; end: gut endoderm; gm: gut mesoderm; dlm: dorsal longitudinal muscles; vlm: ventral longitudinal muscles; vnc: ventral nerve cord; p: parapodia. (i) Ventral view of a 7-day regenerate showing parapodial expression of Pd-WntA. (j) Close up dorsal view of nascent segments in a 7-day regenerate, showing Pd-WntA expression in the walls of lateral vessels (red arrowheads) branching from the dorsal longitudinal vessel (dlv). The yellow dashed line in (e), (g), (i) is the approximate position of the SAZ. [file 1471-2148-10-374-S10.PDF]
